# Biological Activity of Newly Synthesized Benzimidazole and Benzothizole 2,5-Disubstituted Furane Derivatives

**DOI:** 10.3390/molecules26164935

**Published:** 2021-08-14

**Authors:** Livio Racané, Ivo Zlatar, Nataša Perin, Maja Cindrić, Vedrana Radovanović, Mihailo Banjanac, Suresh Shanmugam, Marijana Radić Stojković, Karmen Brajša, Marijana Hranjec

**Affiliations:** 1 Department of Applied Chemistry, Faculty of Textile Technology, University of Zagreb, Prilaz baruna Filipovića 28a, 10000 Zagreb, Croatia; lracane@ttf.hr; 2 Pharmacology In Vitro, Fidelta Ltd., Prilaz baruna Filipovića 29, 10000 Zagreb, Croatia; ivo.zlatar@fidelta.eu (I.Z.); vedrana.radovanovic@fidelta.eu (V.R.); mihailo.banjanac@fidelta.eu (M.B.); 3 Department of Organic Chemistry, Faculty of Chemical Engineering and Technology, University of Zagreb, Marulićev trg 19, 10000 Zagreb, Croatia; nperin@fkit.hr (N.P.); mcindric@fkit.hr (M.C.); 4 Division of Organic Chemistry and Biochemistry, Ruđer Bošković Institute, Bijenička cesta 54, 10000 Zagreb, Croatia; suresh.shanmugam@irb.hr (S.S.); mradic@irb.hr (M.R.S.)

**Keywords:** amidines, acrylonitriles, benzimidazoles, benzothiazoles, furanes, antitumor activity, DNA binding

## Abstract

Newly designed and synthesized cyano, amidino and acrylonitrile 2,5-disubstituted furane derivatives with either benzimidazole/benzothiazole nuclei have been evaluated for antitumor and antimicrobial activity. For potential antitumor activity, the compounds were tested in 2D and 3D cell culture methods on three human lung cancer cell lines, A549, HCC827 and NCI-H358, with MTS cytotoxicity and BrdU proliferation assays in vitro. Compounds **5**, **6**, **8**, **9** and **15** have been proven to be compounds with potential antitumor activity with high potential to stop the proliferation of cells. In general, benzothiazole derivatives were more active in comparison to benzimidazole derivatives. Antimicrobial activity was evaluated with Broth microdilution testing (according to CLSI (Clinical Laboratory Standards Institute) guidelines) on Gram-negative *Escherichia coli* and Gram-positive *Staphylococcus aureus*. Additionally, *Saccharomyces cerevisiae* was included in testing as a eukaryotic model organism. Compounds **5**, **6**, **8**, **9** and **15** showed the most promising antibacterial activity. In general, the compounds showed antitumor activity, higher in 2D assays in comparison with 3D assays, on all three cell lines in both assays. In natural conditions, compounds with such an activity profile (less toxic but still effective against tumor growth) could be promising new antitumor drugs. Some of the tested compounds showed antimicrobial activity. In contrast to ctDNA, the presence of nitro group or chlorine in selected furane-benzothiazole structures did not influence the binding mode with AT-DNA. All compounds dominantly bound inside the minor groove of AT-DNA either in form of monomers or dimer and higher-order aggregates.

## 1. Introduction

Among all known nitrogen heterocycles, benzimidazole and benzothiazole moieties have been recognized as important and well-known constituents of biologically important molecules in medicinal and pharmaceutical chemistry [1,2,3,4,5]. Due to their wide range of biological activities, these heterocycles are still unavoidable structural motifs in the rational design of novel drugs.

Among their versatile pharmacological features, the most important ones are antimicrobial, antitumor, antiviral, anti-inflammatory, antihistaminic, antioxidant, etc. [5,6,7,8]. One of the most commonly used classes of chemotherapeutic agents in medicinal chemistry are still molecules that interact with DNA/RNA [9,10,11,12]. 

**Figure 1:**
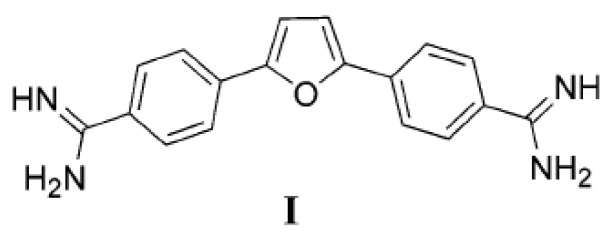
The structure of diamidine furamidine analogue I.

Since amidines are also important structural parts of numerous biologically active compounds, such as many important medical and biochemical agents, suchlike derivatives contribute significantly to the molecule/possible biological target complex stability through H-bonding and electrostatic interactions [13,14]. In several previously published papers we have proven that by incorporation of one or two positively charged amidine moieties at the end of the heteroaromatic benzimidazole/benzothiazole derivatives, we could significantly improve the biological activity [13,14,15,16,17,18,19]. Additionally, the amidine groups allowed and oriented the function of the heteroaromatic derivative toward the binding to an electronegatively charged molecule such as DNA/RNA. For example, we have shown that some benzimidazole and benzothiazole derivatives bearing amidine moiety showed strong antitumor activity by intercalating into ds-RNA and ct-DNA, or by binding into the minor groove of AT-DNA, ss-RNA or showed sequence-selective binding in the A-T rich side of ct-DNA [20,21]. Furthermore, the antimicrobial properties of dicationic molecules with amidine moieties similar to pentamidine have been studied since the beginning of the 20th century, showing activities against a number of pathogens [22,23]. Several suchlike derivatives have been proven to bind to the minor groove of DNA at AT-rich sites [24,25], which was confirmed by different biophysical studies [26] and several crystal structures. Some extensive work has been undertaken in the chemistry of 2,5-disubstituted furane derivatives bearing either one or two amidine moieties. Thus, diamidine furamidine I (Figure 1) showed a broad spectrum of antimicrobial activities [27] while some other similar derivatives have been extensively studied in phase I and/or II of clinical trials. 

The mode of biological action which include the binding to DNA and subsequent inhibition of DNA dependent enzymes has been proposed for these types of molecules [28]. Furthermore, some 2,5-bis(4-amidinophenyl)furan and their substituted amidino derivatives were shown to be effective in vivo at a submicromolar per kilogram body weight dosage against *P. carinii* in the immunosuppressed rat model [29,30]. A group of authors has studied the biological activity of aza-analogues of furamidine by replacing the phenyl ring with pyridine, but none of the derivatives appeared to have significant advantages over the furan analogues [31].

Taking into account that both benzimidazoles and benzothiazoles are among the most privileged heterocyclic subunits and important building blocks in medicinal chemistry, and the important role of the amidino moiety at the end of the heteroaromatic molecules, we have synthesized novel 2,5-disubstituted cyano and amidino furane derivatives. Additionally, since the cyano group has a positive influence on biological activity and acrylonitriles bearing heteroaromatic or aromatic moieties have been recognized as promising biologically active compounds, we have designed and synthesized novel acrylonitrile 2,5-furane derivatives bearing either benzimidazole or benzothiazole nuclei.

All newly designed compounds were tested for screening in vitro on human lung cancer cells on two-dimensional and three-dimensional cell culture assays. 

At a very early stage of the R&D process of new oncology drugs, tests used in the screening phase to identify active molecules with antitumor activity are traditionally performed on tumor cell cultures grown in two-dimensional (2D) conditions that do not provide the same conditions to cells for growth and development as is the case in a living organism. The limitations of such 2D tests are the unnatural shape of the cells (flat), different gene expression, cellular environment, metabolism and other parameters. Therefore, in the last ten years, in vitro cell-based assays have been developed, with more similar conditions to the natural conditions, namely three-dimensional (3D) cultured cell lines, such as morphology, growth and differentiation, metabolic activity and gene expression in comparison with cell grown in 2D cultures [32].

In previous research, we obtained the discrepancy of growth inhibitory concentrations (IC_50_) values of NCE to examine their potential antitumor activity parallel on two-dimensional (2D) and three-dimensional (3D) cell culture assays with different tumor cell lines [33,34]. Therefore, the presented study involves compound testing in 2D and 3D cell cultures on three lung cell lines (A549, HCC827, NCI-H358) in parallel. As a 3D assay model, we used a “hanging-drop” model because the model allows avoidance of NCE interactions with other substrates used as carrier material, and compounds were tested on MTS assay due to determinate antitumor activity and BrdU assay for measurement of their antiproliferative activity. Both tests were performed in parallel in 2D and 3D grown cell cultures and IC_50_ values were compared. As standard compounds, we used three chemotherapeutics (doxorubicin, vandetanib and staurosporine). All compounds were also tested for their antibacterial activity on *E. coli* and *S. aureus* as well as on *S. cerevisiae.*

As mentioned earlier, the study of non-covalent interactions of small molecules with DNA can help in the understanding of their mechanism of action and anticipation of the potential therapeutic significance of such interactions [35]. Estimation of the binding strength and binding mode of small molecule and DNA interactions was performed using UV/Vis, fluorescence and circular dichroism spectroscopy.

## 2. Results and Discussion

### 2.1. Chemistry

**Scheme 1:**
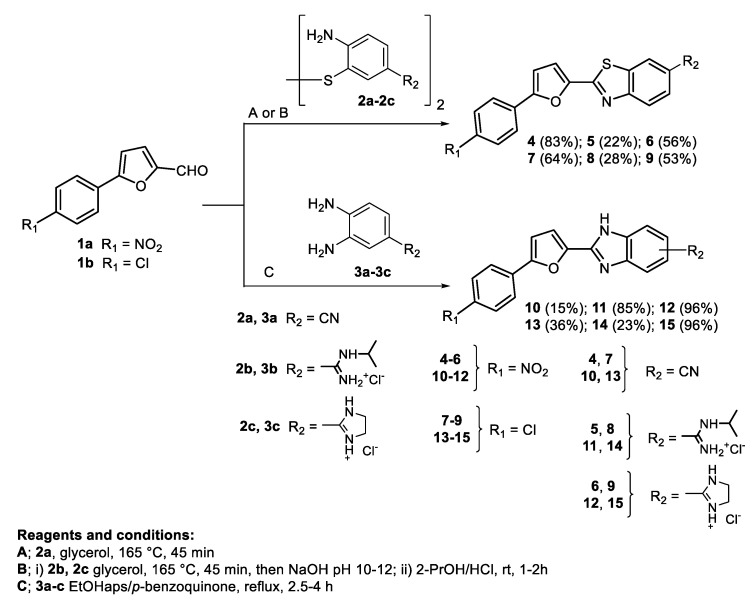
Synthesis of cyano and amidino substituted derivatives **4**–**15**.

**Scheme 2:**
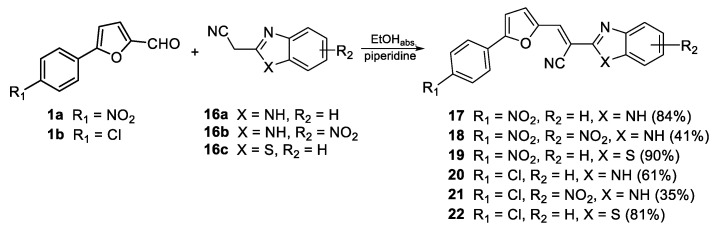
Synthesis of acrylonitrile derivatives **17**–**22**.

The cyano, amidino and acrylonitrile substituted 2,5-furane derivatives bearing benzimidazole or benzothiazole nuclei were synthesized according to the experimental procedures presented in Scheme 1 and Scheme 2. To ensure the efficient synthesis of targeted benzazole derivatives, our well-optimized methods for cyclocondenastion to fused benzazole derivatives were used. Cyano and amidino substituted benzothiazole derivatives **4**–**9** were prepared following the recently developed green synthetic protocol from 2-aminophenyl disulfides and aromatic aldehydes by simple thermal reaction in glycerol, without the need for a catalyst [36]. Applying this condensation method, the corresponding furan-2-carbaldehydes **1a**–**1b** and cyano-substituted 2-aminophenyldisulfide **2a [32]** afforded nitrophenyl- and chlorophenyl- cyanobenzothiazole derivatives **4** and **7** in a very good yield. Condensation of the corresponding amidino-substituted 2-aminophenyldisulfides **2b**–**2c [37]** with aldehydes **1a**–**1b** afforded in a first step the crude free base of 6-(*N*-isopropyl-amidino)benzothiazole and 6-(2-imidazolinyl)benzothiazole derivatives, which in a second step were converted by an excess of hydrochloric acid in 2-propanol in a targeted hydrochlorides **5**–**6** and **8**–**9** in moderate overall reaction yields. Cyano and amidino substituted benzimidazole derivatives **10**–**15** were prepared following the experimental protocol shown in Scheme 1.

Due to the reaction of cyclocondensation of 5-(4-nitro/chlorophenyl) substituted furan-2-carbaldehydes **1a**–**1b** and 4-(cyano/*N*-isopropyl-amidino/4-(2-imidazolinyl)-1,2-phenylene diamine hydrochlorides **2a**–**2c**, corresponding 2,5-furyl-substituted benzimidazoles **10**–**15** were obtained in moderate reaction yields as a mixture of inseparable tautomers.

Amidino substituted 1,2-phenylenediamine **3b**–**3c** were obtained in the acidic Pinner reaction from corresponding cyano substituted precursors according to the previously published procedures [13]. Acrylonitrile substituted 2,5-difurane derivatives bearing either benzimidazole **17**–**18** and **20**–**21** or benzothiazole **19** and **22** nuclei were synthesized according to the procedure shown in Scheme 2 in the reaction of condensation from 5-(4-nitro/chlorophenyl) substituted furan-2-carbaldehydes **1a**–**1b** and 2-cyanomethylbenzimidazole/benzothiazole **16a**–**16c** in absolute ethanol with piperidine as a base [38]. Derivatives **18** and **21** were obtained as a mixture of *E*- and *Z*-isomers.

The structures of all newly prepared cyano, amidino and acrylonitrile substituted benzimidazole/benzothiazole derivatives were confirmed by means of ^1^H and ^13^C NMR spectroscopy as well as elemental analysis. 

NMR analysis based on the values of chemical shifts and H-H coupling constants in the ^1^H spectra confirmed the structures of the compounds. Furthermore, ^13^C NMR chemical shifts additionally confirmed the suggested structures. Additionally, IR spectroscopy was used for the monitoring of the Pinner reaction due to the synthesis of benzimidazole precursors **3b**–**3c**.

### 2.2. Biology

#### 2.2.1. Antiproliferative Activity and Cytotoxicity Assays in 2D and 3D Cell Culture Assays In Vitro

As cytotoxicity and proliferation assays readouts give two distinct characteristics of cells, we tested compounds in MTS cytotoxicity assay and BrdU proliferative assay. MTS assay gives us information about the number of living cells, in this case, metabolically active cells, whereas the BrdU proliferation assay gives a measure of the cell population in active division phase [39]. Both assays were performed in 2D and 3D assay formats. For the 2D cell assay format, we used classic two-dimensional in vitro assays [40] and as a 3D assay we used a hanging drop proliferation cell assay as previously described. We explored the activity of compounds on three human lung cancer cell lines, A549, HCC827 and NCI-H358. The compound activity was also tested on human lung fibroblast cell line MRC-5. A two-dimensional in vitro viability assay was performed on the MRC-5 cell line to see if tested compounds are cytotoxic on normal cell lines in low concentrations. As standard drugs, we use doxorubicin, staurosporine and vandetanib, drugs with different modes of action. Doxorubicin interacts with DNA by intercalation and inhibits the synthesis of biomolecules, staurosporine is a protein kinase C (PKC) inhibitor and vandetanib multikinase inhibitor (VGFRs, EGFR and RET kinase) is an antitumor drug with potential use in a broad range of tumors types, especially thyroid and lung cancer. The results for each of the tested compounds are calculated as growth percentages from two independent concentrations curves compared with the untreated control cells after drug exposure and activity for each compound is presented as an IC_50_ value calculated using the program GraphPadPrism software (La Jolla, CA, USA), v. 7.1.

The obtained IC_50_ values of the tested compounds, as well as for the standard compounds in 2D and 3D cell culture systems in the cytotoxicity MTS assay for each of the three human lung cancer cell lines, are depicted in Table 1. As presented in Table 1, nitro substituted compound **5** bearing *iso*-propyl amidine moiety showed high antitumor activity in 2D assay on A549 and NCI-H358 cell lines and moderate on HCC827. Additionally, compound **5** has moderate cytotoxic activity on healthy MRC-5 lung fibroblasts, which is promising and can be a good start for further molecule optimization. Nitro substituted compound **6** with imidazolinyl group showed high potential antitumor activity in 2D and 3D assay format against all three lung cancer cell cultures (A549 IC_50_, 2.12 ± 0.21 μM and 4.01 ± 0.95 μM; HCC827 IC_50_, 5.13 ± 0.97 μM and 7.02 ± 3.25 μM; NCI-H358 IC_50_ for 0.85 ± 0.05 μM and 1.73 ± 0.01 μM). This compound also affected human lung fibroblast MRC-5 in 2Dcytotoxicity assay (3.11 ± 0.26 μM). Compound **8**, showed more prominent activity against A549 cancer cell line in both assay formats (A548 IC_50_, 6.75 ± 0.19 μM and 9.31 ± 0.78 μM) in comparison to the other tested cell lines. Additionally, in 2D format on the other two cell lines, the activity of chloro substituted derivative **8** was significantly higher (HCC827 IC_50_ 6.26 ± 0.33 μM; NCI-H358 IC_50_ 6.48 ± 0.11 μM), in comparison with the activity in the 3D format assay (HCC827 IC_50_ 20.46 ± 8.63 μM; NCI-H358 IC_50_ 16.00 ± 9.38 μM). The same activity pattern was also shown by the imidazolinyl substituted compounds **9** and **15** (high activity in 2D assay and medium activity in 3D assay). 

The reason that the compounds showed higher activity in 2D than in 3D assay format is expected to be due to how difficult it is for the compounds to penetrate 3D cell spheres and achieve full activity. Compound **20** was non-active on A549 and showed very low activity on other cell lines, except on HCC827 in 3D format assay (9.48 ± 1.15 μM). Compounds, **8** and **9** showed similar activity on the MRC-5 normal lung fibroblast cell line, as could be seen on A549, HCC827 and NCI-H358 cancer cell lines, whereas compound **15** was non-active on MRC-5 cell line compared to three human lung cancer cell lines tested and the compound showed activity in A549, HCC827 and NCI-H358 cancer cell lines at the highest dose.

Active compounds are not selective for tested cancer cell lines, because the normal lung fibroblast cell line MRC-5 is also affected. This may have an impact on further compound development related to dose-limiting acute and chronic toxicities. Therefore, further optimization of the chemical structure of prepared compounds is required. Additionally, the IC_50_ values of control substances (doxorubicin, staurosporine and vandetanib) which showed the same non-selective activity against cancer test lines are presented also in the tables Compound **15** showed very low activity on all cell lines and in both tested formats. All other tested compounds showed no activity in the cytotoxicity assay. If we compare the antiproliferation activity of the compounds presented in Table 2, we can conclude that all compounds, except compound **15**, active in viability assay, also showed antiproliferative activity but in slightly different ways. The most active compound **6** in viability assay also showed the most pronounced antiproliferative activity on all three tested cell lines, (A548 IC_50_, 2.27 ± 0.13 μM and 5.52 ± 0.95 μM; HCC827 (3D) IC_50_ 7.63 ± 5.00 μM; NCI-H358 IC_50_ for 1.10 ± 0.001 μM and 2.36 ± 1.17 μM) except on HCC827 on 2 D format (IC_50_ 16.69 ± 9.39 μM) in which antiproliferative activity was moderate. 

Compound **8** showed high activity against A549 and NCI-H358 cancer cell line in 2D assay formats (A549 IC_50_, 8.78 ± 3.62 μM; NCI-H358 6.68 ± 15 μM) but showed moderate activity in the 3D format assay (A548 IC_50_, 19.94 ± 2.19 μM; NCI-H358 11.27 ± 0.49 μM).

Additionally, antiproliferative activity was moderate for HCC827 for both assays formats (IC_50_ 13.48 ± 0.81 μM and 17.75 ± 4.24 μM). Compounds **5** and **9** showed the same range and pattern of activity as in the viability assay (higher activity in 2D in comparison to 3D format assay) with the exception of NCI-H358 in 3D assay format, in which **9** showed a shift of activity from moderate activity in viability assay (IC50 15.49 ± 0.07 μM) to high activity in the antiproliferative assay (IC_50_ 6.39 ± 3.29 μM). Compound **20** was again non-active on A549, showed moderate activity on other cell lines and high activity on HCC827 in the 3D format assay (0.16 ± 0.11 μM) as in viability assay. Interestingly, besides **15**, which showed very low activity on all cell lines and in both tested formats in the viability test and was not active in the proliferative assay (except in NCI-H358, IC_50_ 20.08 ± 2.82 μM), compounds **13** and **14**, additionally showed low antiproliferative activity in the NCI-H358 cell line in 2D assay format (IC_50_ 15.67 ± 0.86 μM; IC_50_ 20.20 ± 0.71 μM). All other tested compounds showed no activity in the proliferative assay. The standard compounds showed the expected activity according to our previous experiments and the literature data. It is very difficult to compare IC_50_ values with the literature data, especially for 3D cultures, because the amount of such data is very limited and assay condition parameters are various.

In conclusion, 2,5-disubstituted benzothiazole derivatives **4**–**9** have more pronounced antiproliferative activity when compared to their benzimidazole analogues **10**–**15**. The most active compounds have proven to be both nitro **5** and chloro **8** substituted benzothiazole derivatives bearing *iso*-propyl amidine, as well as benzothiazoles **6** and **9** bearing the imidazolinyl group. Furthermore, imidazolinyl substituted benzothiazole derivatives **5**–**6** were more active in comparison to their *iso*-propyl amidine substituted analogues **8**–**9**. Additionally, the nitro group in comparison to chlorine substituents have improved antiproliferative activity. Regarding the 2,5-disubstituted benzimidazole derivatives **10**–**15**, the most active was cyano substituted derivative **13**, while nitro substituted derivative **12** bearing the imidazolinyl group showed selective activity towards HCC827 cells. Among the acrylonitrile derivatives, the most pronounced activity was observed within the chloro substituted benzimidazole derivative **20**, with selective activity against HCC827 and NCI-H358 cells.

Therefore, it is always important to have standard compounds in every test run and observed quality control parameters of each growth curve. Due to the well-known fact that the 2,5-disubstituted furane derivatives are also potential antimicrobial agents, we tested the prepared derivatives on a primary antibacterial screening panel which includes *E. coli* ATCC 25,922 and *S. aureus* ATCC 29213. The summarized results are presented in Table 3. Only compound **15** showed significant antimicrobial activity against *S. aureus* (MIC 6.12 μM) and moderate activity against *E. coli* (MIC 25 μM). Both amidino substituted benzothiazole derivatives **8**, **9** and *iso*-propyl amidino substituted benzimidazole **14** showed moderate activity to *S. aureus* (MIC 12.5, 25, 12.5 μM). As we expected, the compounds that showed activity in MTS assay on eukaryotic cell lines were active against *S. cerevisiae* (**5**, **6**, **8**, **9** and **15**).

#### 2.2.2. DNA Binding Study

Based upon the antiproliferative activity, compounds **5**, **6**, **8**, **9** and **15** were selected for the binding study with *calf thymus* DNA (ctDNA), which contains 58% AT and 42% GC nucleobases and represents a classical B-helix [41]. Polynucleotide addition to compound solutions resulted in an emission decrease in all selected compounds (Figure S5, Supporting information (SI)). The estimated binding constants *Ks* obtained by processing of fluorimetric titration data were of the same order of magnitude for all complexes of studied compounds with ctDNA (log *Ks* ≈ 6, Table S2, SI) [42].

The thermal stability of helices is usually affected by the non-covalent binding of small molecules to double-stranded nucleic acids [43]. We determined the Δ*T*_m_ value (the difference between the *T*_m_ value of free polynucleotide and a complex with a small molecule) for complexes of selected compounds with ctDNA and an alternating polynucleotide, poly(dAdT)_2_. All studied compounds showed a greater stabilization effect of poly(dAdT)_2_ compared to mixed base pair composition, ctDNA (Table 4). Because of this, we determined Δ*T*_m_ value with non-alternating AT-DNA, poly dA-poly dT as well.

**Figure 2:**
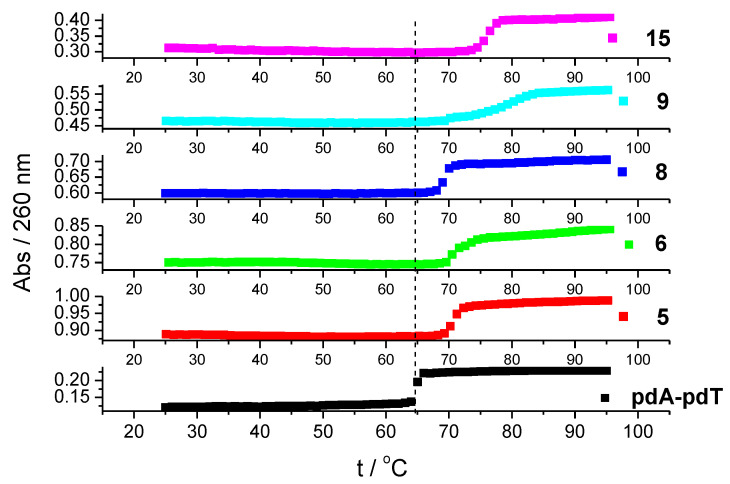
Melting curve of poly dA-poly dT upon addition of ratio, *r* ([compound]/[polynucleotide]) = 0.3 of **5, 6, 8, 9** and **15** at pH = 7.0 (buffer sodium cacodylate, *I* = 0.05 mol dm^−3^).

All compounds showed better stabilization effects with consecutive AT nucleobases, poly dA-poly dT than with poly(dAdT)_2_ (Figure 2). Such preference toward AT basepairs is indicative of minor groove binders [44].

**Figure 3:**
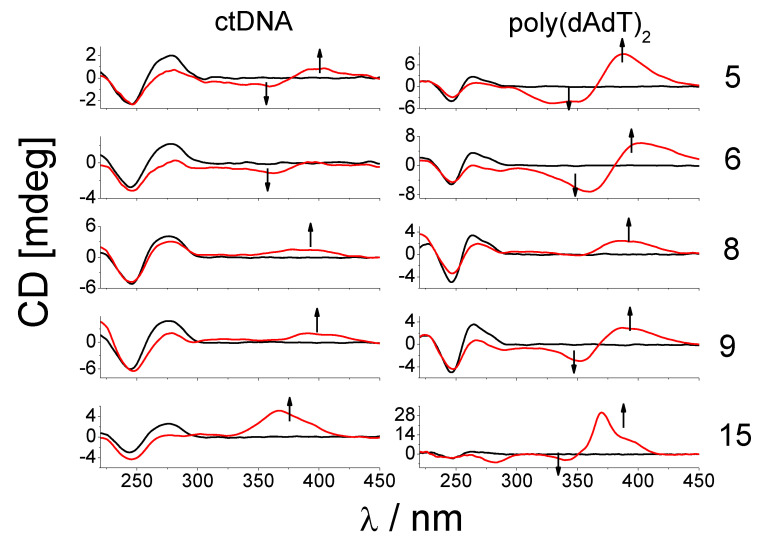
CD titrations of ctDNA and poly(dAdT)_2_ (*c* = 3.0 × 10^−5^ mol dm^−3^) with **5_,_ 6_,_ 8_,_ 9** and **15** at molar ratios ***r*** = [compound]/[polynucleotide] = 0.5; (polynucleotide ─, complex ─), pH = 7.0, buffer sodium cacodylate, *I* = 0.05 mol dm^−3^.

CD spectroscopy is often utilized for monitoring the specific signature of secondary structures of nucleic acids and structural changes resulting from interactions with various ligands or changes in conditions such as ionic strength, pH and temperature [45]. Induced CD spectra (ICD) produced after the binding of achiral ligands to nucleic acids may be particularly informative in assessing the modes of binding (intercalation, groove binding) [46]. The addition of compounds **8**, **9** and **15** caused a decrease in CD spectra of ctDNA at 275 nm and strong positive ICD bands (360–400 nm) that suggest minor groove binding to DNA. The binding of compounds **5** and **6** to ctDNA caused the appearance of bisignate ICD signals (360–400 nm) and negative ICD signals (305–360 nm), respectively (Figure 3 and Figure S8, SI).

Such changes in CD spectra suggest the binding of **5** inside the ctDNA groove, most probably in the form of a dimer. Negative ICD signals in a titration of **6** are indicative of intercalative binding where the ligand is oriented “parallel” to the long axis of adjacent base pairs. At ratios ***r*** higher than 0.3, compound **6** probably form aggregates along the DNA backbone [47].

The nitro group has a stronger electron-withdrawing effect on a given compound than chlorine. This could be the reason for different modes of binding of furane-benzothiazole derivatives with the nitro group and chlorine to ctDNA. However, the assessment of the substituent effects of both groups on the electronic properties of the rest of the molecule would require a quantitative description of changes in the electronic structure of these systems, which exceeds the scope of this work [48]. Surprisingly, almost all derivatives, regardless of whether they have a chlorine or nitro group in their structure, induced the formation of bisignate signals in the CD spectra of poly(dAdT)_2_ (range 330–400 nm).

These changes can probably be attributed to the binding of compound dimer or higher-order aggregates inside of poly(dAdT)_2_ groove or along the polynucleotide backbone. Only compound **8** exhibited similar changes with poly(dAdT)_2_ as with ctDNA. 

The most likely reason for this is a different amidine moiety on the benzazole heterocycle; while compounds **9** and **15** have imidazole moiety, **8** possesses isopropylamidino moiety that probably interferes with dimer formation.

In addition, only at the lowest ratio, r = 0.1, did compounds **9** and **15** induce changes in the CD spectra of poly(dAdT)_2_ (significant positive ICD signals at 390 and 370 nm, respectively) such as with ctDNA.

Interestingly, compound **15**, which differs from compound **9** only by benzazole ring (benzimidazole **15** vs. benzothiazole **9**) caused the largest changes in the CD spectra of poly(dAdT)_2_, a significant decrease in CD intensity of DNA at 262 nm, an appearance of strong negative CD signal at 282 nm and positive/negative CD bands with maxima at 340 and 368 nm (Figure 3 and Figure S8, SI).

**Figure 4:**
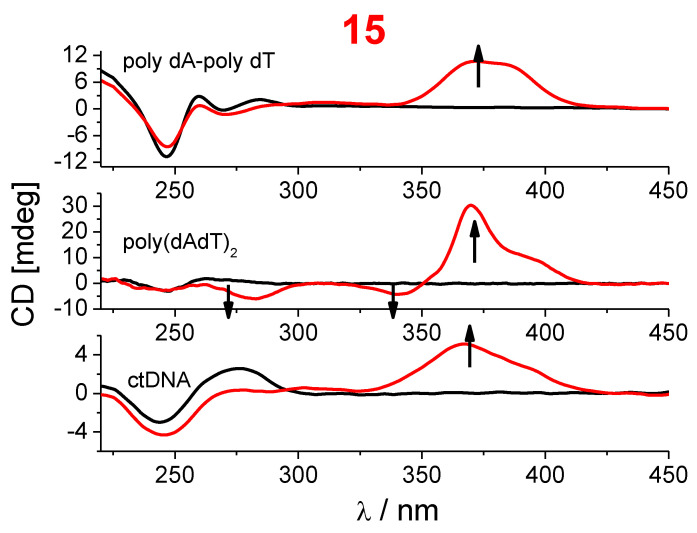
CD titrations of ctDNA, poly(dAdT)_2_ and poly dA- poly dT (*c* = 3.0 × 10^−5^ mol dm^−3^) with **15** at molar ratios ***r*** = [compound]/[polynucleotide] = 0.5; (polynucleotide ─, complex ─), pH = 7.0, buffer sodium cacodylate, *I* = 0.05 mol dm^−3^.

The zero-crossing point of the unsymmetric bisignate CD signal was at 352 nm, in good agreement with the absorption maximum of **15** (Figure 3 and Figure S2, SI). The greatest magnitude of bisignate CD signal was at the ratio ***r*** = 0.3. A strong decrease in CD intensity of poly(dAdT)_2_ in the range from 260 to 290 nm was probably not a consequence of the significant changes of the polynucleotide conformation but rather a uniform orientation of benzimidazole-furan chromophore transition moments with regard to DNA chiral axis which resulted in a negative ICD spectrum in this range. Due to strong changes in CD spectra of the alternating AT copolymer, poly(dAdT)_2_ with **15**, we performed CD titration with non-alternating AT polynucleotide, poly dA-poly dT (Figure 4). The decrease in CD intensity of poly dA-poly dT at their maxima at 260 at 283 nm was smaller than with alternating AT polynucleotide. Unlike with alternating AT-polynucleotide, the addition of **15** to poly dA-poly dT solution did not cause the appearance of bisignate ICD signal but a significant positive ICD signal in the range from 345 to 408 nm, similar to the ICD signal obtained with ctDNA (Figure 4).

Although both of these polynucleotides belong to the B-DNA family, non-alternating poly dA-poly dT differs from a classical B-helix by a distinct secondary B structure characterized by a much narrower and deeper minor groove than that of poly(dAdT)_2_ [49]. Such differentiation between ICD signals (range from 345–405 nm) of alternating and non-alternating AT-copolymers is probably the consequence of different binding possibilities inside the minor grooves (extent of hydrophobic interactions, the difference in electrostatic molecular potential). Most likely, compound **15** binds in the form of dimer to alternating poly(dAdT)_2_ and in the form of a monomer inside the narrower minor groove of non-alternating poly dA-poly dT.

## 3. Materials and Methods

### 3.1. Chemistry

#### 3.1.1. General

Melting points were determined by means of Original Kofler Mikroheitztisch apparatus (Reichert, Wien). ^1^H NMR and^13^C NMR spectra were recorded with the Bruker Avance DPX-300 or Bruker AV-600 using TMS as an internal standard. Chemical shifts are reported in parts per million (ppm) relative to TMS.

Elemental analyses for carbon, hydrogen and nitrogen were performed on Perkin-Elmer 2400 elemental analyzer. Analyses are indicated as symbols of elements, and the analytical results obtained are within 0.4% of the theoretical value.

#### 3.1.2. General Method for Preparation of Compounds **4** and **7**

To a stirred suspension of disulfide **2a** (0.5 eq) in glycerol (1–1.5 g), the corresponding aldehyde **1a**–**1b** (1.0 eq) was added and heated at 165 °C for 45 min. The reaction mixture was cooled below 100 °C, quenched with ethanol and cooled in the refrigerator overnight. The resulting precipitate was collected by filtration, washed with ethanol and air-dried.

*6-Cyano-2-[5-(4-nitrophenyl)furan-2-yl]benzothiazole **4***.

Compound **4** was prepared from 5-(4-nitrophenyl)furan-2-carbaldehyde **1a** (0.109 g, 0.5 mmol) and bis(2-amino-5-cyanophenyl) disulfide **2a** (0.075 g, 0.25 mmol) to yield 0.144 g (83%) of beige powder; m.p. = 276–279 °C. ^1^H NMR (600 MHz, DMSO-*d*_6_) δ = 8.78 (d, *J* = 1.4 Hz, 1H, Ar-H), 8.36 (d, *J* = 8.9 Hz, 2H, Ar-H), 8.20 (d, *J* = 8.3 Hz, 1H, Ar-H), 8.12 (d, *J* = 8.8 Hz, 2H, Ar-H), 7.96 (dd, *J* = 8.4, 1.5 Hz, 1H, Ar-H), 7.70 (d, *J* = 3.8 Hz, 1H, Ar-H), 7.64 (d, *J* = 3.8 Hz, 1H, Ar-H). Anal. calc. for C_18_H_10_N_3_O_3_S: C, 62.24; H, 2.61; N, 12.10. Found: C, 62.13; H, 2.67; N, 12.04.

*2-[5-(4-Chlorophenyl)furan-2-yl]-6-cyanobenzothiazole **7***.

Compound **7** was prepared from 5-(4-chlorophenyl)furan-2-carbaldehyde **1b** (0.104 g, 0.5 mmol) and bis(2-amino-5-cyanophenyl) disulfide **2a** (0.075 g, 0.25 mmol) to yield 0.108 g (64%) of yellow powder; m.p. = 235–237 °C. ^1^H NMR (600 MHz, DMSO-*d*_6_) δ = 8.75 (d, *J* = 1.4 Hz, 1H, Ar-H), 8.17 (d, *J* = 8.4 Hz, 1H, Ar-H), 7.95 (dd, *J* = 8.4, 1.6 Hz, 1H, Ar-H), 7.90 (d, *J* = 8.6 Hz, 2H, Ar-H), 7.63 (d, *J* = 3.7 Hz, 1H, Ar-H), 7.59 (d, *J* = 8.6 Hz, 2H, Ar-H), 7.38 (d, *J* = 3.7 Hz, 1H, Ar-H). ^13^C NMR (150 MHz, DMSO-*d*_6_) δ = 160.4, 156, 155.4, 147.2, 134.5, 133.6, 130.0, 129.2 (2C), 127.8, 127.5, 126.1 (2C), 123.3, 118.6, 116.1, 109.8, 107.5. Anal. calc. for C_18_H_9_ClN_2_OS: C, 64.19; H, 2.69; N, 8.32. Found: C, 64.31; H, 2.60; N, 8.30.

#### 3.1.3. General Method for Preparation of Compounds **5**–**6** and **8**–**9**

To a stirred suspension of disulfides **2b**–**2c** (0.5 eq) in glycerol (1.5 g), the corresponding aldehyde **1a**–**1b** (1.0 eq) was added and heated at 165 °C for 45 min. The reaction mixture was cooled, diluted with water and made alkaline (pH 10–12) with 20% NaOH. The resulting free base was filtered, washed with water and dried. 

The crude free base was suspended in 2-propanol and an excess of concentrated hydrochloric acid was added and stirred at room temperature for 1–2 h. 

*2-[5-(4-Nitrophenyl)furan-2-yl]-6-N-isopropilamidinobenzothiazole hydrochloride **5***.

Compound **5** was prepared from 5-(4-nitrophenyl)furan-2-carbaldehyde **1a** (0.098 g, 0.45 mmol) and bis(2-amino-5-*N*-isopropilamidinophenyl) disulfide dihydrochloride dihydrate **2b** (0.112 g, 0.23 mmol). The obtained free base was suspended in 2-propanol (4 mL) and concentrated hydrochloric acid (0.125 mL) was added and stirred at room temperature for 1 h. After cooling overnight, the resulting precipitate was filtered, washed with diethyl-ether to yield 0.043 g (22%) of pale orange powder; m.p. = 201–206 °C. ^1^H NMR (300 MHz, DMSO-*d*_6_) δ = 9.44 (bs, 3H, NH_amidine_), 8.63 (d, *J* = 1.6 Hz, 1H, Ar-H), 8.38 (d, *J* = 8.9 Hz, 2H, Ar-H), 8.25 (d, *J* = 8.6 Hz, 1H, Ar-H), 8.15 (d, *J* = 8.9 Hz, 2H, Ar-H), 7.87 (dd, *J* = 8.6, 1.7 Hz, 1H, Ar-H), 7.71 (d, *J* = 3.7 Hz, 1H, Ar-H), 7.67 (d, *J* = 3.8 Hz, 1H, Ar-H), 4.13–4.03 (m, 1H, CH), 1.31 (d, *J* = 6.3 Hz, 6H, CH_3_). ^13^C NMR (75 MHz, DMSO-*d*_6_) δ = 161.3, 159.4, 155.9, 153.8, 148.5, 146.8, 134.5, 133.9, 126.9, 126.5, 125.1 (2C), 124.6 (2C), 123.5, 122.7, 116.1, 113.1, 45.2, 21.4 (2C). Anal. calc. for C_21_H_19_ClN_4_O_3_S: C, 56.95; H, 4.32; N, 12.65. Found: C, 57.03; H, 4.28; N, 12.61.

*6-(4,5-Dihydro-1H-imidazol-2-yl)-2-[5-(4-nitrophenyl)furan-2-yl]benzothiazole hydrochloride **6***.

Compound **6** was prepared from 5-(4-nitrophenyl)furan-2-carbaldehyde **1a** (0.110 g, 0.50 mmol) and bis [2-amino-5-(4,5-Dihydro-1*H*-imidazol-2-yl)phenyl] disulfide dihydrochloride dihydrate **2c** (0.123 g, 0.25 mmol). The obtained free base was suspended in 2-propanol (15 mL) and concentrated hydrochloric acid (0.150 mL) was added and stirred at room temperature for 2 h. After cooling overnight, the resulting precipitate was filtered, washed with diethyl-ether to yield 0.120 g (56%) of pale orange powder; m.p. = 287–292 °C. ^1^H NMR (300 MHz, DMSO-*d*_6_) δ = 10.82 (s, 2H, NH_amidine_), 8.86 (d, *J* = 1.5 Hz, 1H, Ar-H), 8.36 (d, *J* = 8.9 Hz, 2H, Ar-H), 8.29 (d, *J* = 8.5 Hz, 1H, Ar-H), 8.16–8.10 (m, 3H, Ar-H), 7.72 (d, *J* = 3.7 Hz, 1H, Ar-H), 7.66 (d, *J* = 3.7 Hz, 1H, Ar-H), 4.06 (s, 4H, CH_2_). Anal. calc. for C_20_H_15_ClN_4_O_3_S: C, 56.27; H, 3.54; N, 13.12. Found: C, 56.20; H, 3.67; N, 13.18.

*2-[5-(4-Chlorophenyl)furan-2-yl]-6-N-isopropilamidinobenzothiazole hydrochloride **8***.

Compound **8** was prepared from 5-(4-chlorophenyl)furan-2-carbaldehyde **1b** (0.103 g, 0.5 mmol) and bis(2-amino-5-*N*-isopropilamidinophenyl) disulfide dihydrochloride dihydrate **2b** (0.122 g, 0.25 mmol). The obtained free base was suspended in 2-propanol (3 mL) and concentrated hydrochloric acid (0.100 mL) was added and stirred at room temperature for 2 h. After cooling overnight, the resulting precipitate was filtered, washed with diethyl-ether to yield 0.061 g (28%) of yellow powder; m.p. = 204–208 °C. ^1^H NMR (300 MHz, DMSO-*d*_6_) δ = 9.84–9.40 (m, 3H, NH_amidine_), 8.64 (d, *J* = 1.5 Hz, 1H, Ar-H), 8.20 (d, *J* = 8.6 Hz, 1H, Ar-H), 7.92–7.86 (m, 3H, Ar-H), 7.62–7.57 (m, 3H, 3H_,_ Ar-H), 7.40 (d, *J* = 3.6 Hz, 1H, Ar-H), 4.26–4.14 (m, 1H, CH), 1.32 (d, *J* = 6.3 Hz, 6H, CH_3_). ^13^C NMR (75 MHz, DMSO-*d*_6_) δ = 161.4, 159.7, 156.1, 155.1, 147.2, 133.8, 133.5, 129.3 (2C), 127.7, 126.9, 126.0 (2C), 125.9, 123.5, 122.4, 116.0, 110.0, 45.3, 21.3 (2C). Anal. calc. for C_21_H_19_Cl_2_N_3_OS: C, 58.34; H, 4.43; N, 9.72. Found: C, 58.38; H, 4.38; N, 9.72.

*2-[5-(4-Chlorophenyl)furan-2-yl]-6-(4,5-Dihydro-1H-imidazol-2-yl)benzothiazole hydrochloride **9***.

Compound **9** was prepared from 5-(4-chlorophenyl)furan-2-carbaldehyde **1b** (0.103 g, 0.5 mmol) and bis[2-amino-5-(4,5-Dihydro-1*H*-imidazol-2-yl)phenyl] disulfide dihydrochloride dihydrate **2c** (0.123 g, 0.25 mmol). The obtained free base was suspended in 2-propanol (10 mL) and concentrated hydrochloric acid (0.125 mL) was added and stirred at room temperature for 1 h. After cooling overnight, the resulting precipitate was filtered, washed with diethyl-ether to yield 0.110 g (53%) of yellow powder powder; m.p. = 293–297 °C. ^1^H NMR (600 MHz, DMSO-*d*_6_) δ = 10.87 (s, 2H, NH_amidine_), 8.88 (d, *J* = 1.6 Hz, 1H, Ar-H), 8.25 (d, *J* = 8.5 Hz, 1H, Ar-H), 8.14 (dd, *J* = 8.6, 1.7 Hz, 1H, Ar-H), 7.91 (d, *J* = 8.6 Hz, 2H, Ar-H), 7.64 (d, *J* = 3.7 Hz, 1H, Ar-H), 7.59 (d, *J* = 8.5 Hz, 2H, Ar-H), 7.40 (d, *J* = 3.7 Hz, 1H, Ar-H), 4.05 (s, 4H, CH_2_). ^13^C NMR (75 MHz, DMSO-*d*_6_) δ = 164.2, 160.3, 156.8, 155.3, 146.9, 134.1, 133.5, 129.2 (2C), 127.5, 126.9, 125.9 (2C), 123.7, 122.8, 118.5, 116.3, 110.0, 44.4 (2C). Anal. calc. for C_20_H_15_Cl_2_N_3_OS: C, 57.70; H, 3.63; N, 10.09. Found: C, 57.63; H, 3.65; N, 10.18.

#### 3.1.4. General Method for Preparation of Compounds **10**–**15**

Solution of equimolar amounts of aldehydes **1a**–**1b**, corresponding derivatives **3a**–**3c**, and *p*-benzoquinone in absolute ethanol, was refluxed for 2.5–4 h. After the reaction mixture was cooled to room temperature, the crude product was filtered off and washed with diethyl ether or was separated by column chromatography on SiO_2_.

*5(6)-Cyano-2-[5-(4-nitrophenyl)furan-2-yl]benzimidazole **10***.

Compound **10** was prepared from 5-(4-nitrophenyl)furan-2-carbaldehyde **1a** (0.60 g, 2.70 mmol), 3,4-diaminobenzonitrile **3a** (0.37 g, 2.70 mmol) and *p*-benzoquinone (0.29 g, 2.70 mmol) in absolute ethanol (15 mL) after refluxing for 4 h. Resulting product was separated by column chromatography on SiO_2_ using dichloromethane as eluent to yield 0.15 g (15%) of red powder; m.p. 242–246 °C. ^1^H NMR (600 MHz, DMSO-*d*_6_) δ = 8.64 (s, 1H, Ar-H), 8.34 (d, *J* = 9.0 Hz, 2H, Ar-H), 8.13 (d, *J* = 9.0 Hz, 2H, Ar-H), 7.58 (d, *J* = 1.7 Hz, 1H, Ar-H), 7.54 (d, *J* = 3.7 Hz, 1H, Ar-H), 7.38 (d, *J* = 3.7 Hz, 1H, Ar-H), 7.35 (dd, *J* = 8.4, 1.8 Hz, 1H, Ar-H). ^13^C NMR (75 MHz, DMSO-*d*_6_) δ = 153.9, 149.0, 147.1, 146.8, 135.5, 135.0, 132.6, 132.2, 125.5, 125.0, 121.2, 120.8, 119.6, 114.8, 113.1, 96.6. Found: Anal. calc. for C_18_H_10_N_4_O_3_: C, 65.45; H, 3.05; N, 16.96. Found: C, 65.55; H, 3.08; N, 16.95.

*2-[5-(4-Nitrophenyl)furan-2-yl]-5(6)-N-isopropilamidinobenzimidazole hydrochloride **11***.

Compound **11** was prepared from 5-(4-nitrophenyl)furan-2-carbaldehyde **1a** (0.30 g, 1.40 mmol), 4-(*N*-isopropylamidino)-1,2-phenylenediamine hydrochloride **3b** (0.32 g, 1.40 mmol) and *p*-benzoquinone (0.15 g, 1.40 mmol) in absolute ethanol (15 mL) after refluxing for 2.5 h to yield 0.49 g (85%) of dark yellow powder; m.p. > 270 °C. ^1^H NMR (300 MHz, DMSO-*d*_6_) δ = 13.88 (s, 1H, NH_benzim._), 9.56 (s, 2H, NH_amidine_), 9.42 (s, 1H, NH_amidine_), 9.01 (s, 1H, Ar-H), 8.39 (d, *J* = 8.9 Hz, 2H, Ar-H), 8.22 (d, *J* = 8.9 Hz, 2H, Ar-H), 7.87–7.76 (m, 1H, Ar-H), 7.61–7.56 (m, 3H, Ar-H), 4.11–4.05 (m, 1H, NH), 1.31 (d, *J* = 6.3 Hz, 6H, 2CH_3_). ^13^C NMR (150 MHz, DMSO-*d*_6_) δ = 162.4, 146.5, 146.0, 135.1, 124.8, 124.5, 112.5, 45.0, 21.3 (2C). Anal. calc. for C_21_H_20_ClN_5_O_3_: C, 59.23; H, 4.73; N, 16.44. Found: C, 59.22; H, 4.75; N, 16.42.

*5(6)-(4,5-Dihydro-1H-imidazol-2-yl)-2-[5-(4-nitrophenyl)furan-2-yl]benzimidazole hydrochloride **12***.

Compound **12** was prepared from 5-(4-nitrophenyl)furan-2-carbaldehyde **1a** (0.30 g, 1.40 mmol), 4-(4,5-dihydro-1*H*-imidazol-2-yl)-1,2-phenylenediamine hydrochloride **3c** (0.33 g, 1.40 mmol) and *p*-benzoquinone (0.29 g, 2.70 mmol) in absolute ethanol (10 mL) after refluxing for 3 h to yield 0.54 g (96%) of dark green powder; m.p. > 270 °C. ^1^H NMR (300 MHz, DMSO-*d*_6_) δ = 14.05 (s, 1H, NH_benzim._), 13.94 (s, 1H, NH_benzim._), 10.66 (s, 2H, NH_amidine_), 10.61 (s, 2H, NH_amidine_), 8.37 (d, *J* = 8.9 Hz, 2H, Ar-H), 8.33–8.27 (m, 1H, Ar-H), 8.21 (d, *J* = 8.9 Hz, 1H, Ar-H), 8.17–8.10 (m, 1H, Ar-H), 7.96–7.78 (m, 4H, Ar-H), 7.63–7.53 (m, 4H, Ar-H), 4.04 (s, 8H, CH_2_). ^13^C NMR (75 MHz, DMSO-*d*_6_) δ = 146.5, 145.7, 135.0, 124.9, 124.8 (2C), 124.5 (2C), 124.3, 123.8, 115.6, 112.5, 44.21 (2C). Anal. calc. for C_20_H_16_ClN_5_O_3_: C, 58.61; H, 3.94; N, 17.09. Found: C, 58.44; H, 3.99; N, 17.43.

*2-[5-(4-Chlorophenyl)furan-2-yl]-5(6)-cyanobenzimidazole **13***.

Compound **13** was prepared from 5-(4-chlorophenyl)furan-2-carbaldehyde **1b** (0.50 g, 2.40 mmol), 3,4-diaminobenzonitrile **3a** (0.32 g, 2.40 mmol) and *p*-benzoquinone (0.26 g, 2.40 mmol) in absolute ethanol (10 mL) after refluxing for 3 h to yield 0.27 g (36%) of green powder; m.p. 242–246 °C. ^1^H NMR (300 MHz, DMSO-*d*_6_) δ = 13.57 (bs, 1H, NH_benzim._), 8.58 (s, 1H, Ar-H), 7.97 (d, *J* = 8.4 Hz, 2H, Ar-H), 7.90 (d, *J* = 8.6 Hz, 2H, Ar-H), 7.61–7.54 (m, 4H, Ar-H). ^13^C NMR (150 MHz, DMSO-*d*_6_) δ = 144.1, 132.9, 129.3, 129.1, 129.1 (2C), 128.2, 126.7, 125.8 (2C), 119.9, 115.6, 109.2, 107.4, 104.1. Anal. calc. for C_18_H_10_ClN_3_O: C, 67.61; H, 3.15; N, 13.14. Found: C, 67.60; H, 3.13; N, 13.17.

*2-[5-(4-Chlorophenyl)furan-2-yl]-5(6)-isopropilamidinobenzimidazole hydrochloride **14***.

Compound **14** was prepared from 5-(4-chlorophenyl)furan-2-carbaldehyde **1b** (0.30 g, 1.40 mmol), 4-(*N*-isopropylamidino)-1,2-phenylenediamine hydrochloride **3b** (0.32 g, 1.40 mmol) and *p*-benzoquinone (0.15 g, 1.40 mmol) in absolute ethanol (10 mL) after refluxing for 3 h to yield 0.13 g (23%) of light grey powder; m.p. 242–246 °C. ^1^H NMR (300 MHz, DMSO-*d*_6_) δ = 13.73 (bs, 1H, NH_benzim._), 9.48 (bs, 3H, NH_amidine_), 8.00 (s, 1H, Ar-H), 7.98 (d, *J* = 8.5 Hz, 2H, Ar-H, 7.78 (d, *J* = 8.3 Hz, 1H, Ar-H), 7.60 (d, *J* = 8.7 Hz, 2H, Ar-H), 7.56 (d, *J* = 8.6 Hz, 1H, Ar-H), 7.47 (d, *J* = 3.6 Hz, 1H, Ar-H), 7.32 (d, *J* = 3.6 Hz, 1H, Ar-H), 4.15–4.02 (m, 1H, CH), 1.31 (d, *J* = 6.3 Hz, 6H, CH_3_). ^13^C NMR (75 MHz, DMSO-*d*_6_) δ = 162.9, 154.2, 144.9, 133.3, 129.6 (2C), 128.7, 126.3 (2C), 123.4, 114.8, 109.6, 45.5, 21.8 (2C). Anal. calc. for C_21_H_20_Cl_2_N_4_O: C, 60.73; H, 4.85; N, 13.49. Found: C, 60.72; H, 4.87; N, 13.48.

*2-[5-(4-Chlorophenyl)furan-2-yl]-5(6)-(4,5-dihydro-1H-imidazol-2-yl)benzimidazole hydrochloride **15***.

Compound **15** was prepared from 5-(4-chlorophenyl)furan-2-carbaldehyde **1b** (0.30 g, 1.40 mmol), 4-(4,5-dihydro-1*H*-imidazol-2-yl)-1,2-phenylenediamine hydrochloride **3c** (0.34 g, 1.40 mmol) and *p*-benzoquinone (0.15 g, 1.40 mmol) in absolute ethanol (10 mL) after refluxing for 3 h to yield 0.54 g (96%) of grey powder; m.p. 262–269 °C. ^1^H NMR (300 MHz, DMSO-*d*_6_) δ = 10.83 (bs, 2H, NH_amidine_), 8.35 (s, 1H, Ar-H), 7.99 (d, *J* = 8.6 Hz, 2H, Ar-H), 7.87 (d, *J* = 8.6 Hz, 1H, Ar-H), 7.81 (d, *J* = 8.5 Hz, 1H, Ar-H), 7.60 (d, *J* = 8.6 Hz, 2H, Ar-H), 7.50 (d, *J* = 3.6 Hz, 1H, Ar-H), 7.32 (d, *J* = 3.6 Hz, 1H, Ar-H), 4.03 (s, 4H, CH_2_). ^13^C NMR (75 MHz, DMSO-*d*_6_) δ = 165.7, 154.4, 144.7, 133.4, 129.5 (2C), 128.7, 126.3 (2C), 123.2, 116.1, 116.0, 115.1, 109.6 (2C), 44.7 (2C). Anal. calc. for C_20_H_16_Cl_2_N_4_O: C, 60.16; H, 4.04; N, 14.03. Found: C, 60.18; H, 4.03; N, 14.04.

#### 3.1.5. General Method for Preparation of Compounds **17**–**22**

Solution of equimolar amounts of heteroaromatic aldehydes **1a**–**1b**, the corresponding derivatives **16a**−**16c**, and a few drops of piperidine in absolute ethanol, was refluxed for 1 h. After the reaction mixture was cooled to room temperature, the crude product was filtered off and recrystallized from ethanol or was separated by column chromatography on SiO_2_.

*(E)-2-(Benzimidazol-2-yl)-3-[5-(4-nitrophenyl)furan-2-yl]acrylonitrile **17 [50]***.

Compound **17** was prepared from 5-(4-nitrophenyl)furan-2-carbaldehyde **1a** (1.38 g, 6.00 mol) and 2-cyanomethylbenzimidazole **16a** (1.00 g, 6.00 mol) in absolute ethanol (10 mL) after refluxing for 2 h. Resulting product was filtered off and recrystallized from ethanol to yield 1.78 g (84%) of orange powder; m.p. 273–274 °C. ^1^H NMR (300 MHz, DMSO-*d*_6_) δ = 13.10 (s, 1H, NH_benzim._), 8.39 (d, 2H, *J* = 8.9 Hz, Ar-H), 8.20 (s, 1H, Ar-H), 8.14 (d, 2H, *J* = 8.8 Hz, Ar-H), 7.69 (d, 1H, *J* = 8.7 Hz, Ar-H), 7.68 (d, 1H, *J* = 3.7 Hz, Ar-H), 7.54 (d, 1H, *J* = 8.7 Hz, Ar-H), 7.46 (d, 1H, *J* = 3.8 Hz, Ar-H), 7.30–7.23 (m, 2H, Ar-H). ^13^C NMR (75 MHz, DMSO-*d*_6_) δ = 154.9, 150.4, 147.7, 147.4, 144.0, 135.4, 135.0, 130.0, 125.7, 125.1, 124.2, 122.8, 119.6, 116.8, 113.8, 112.0, 99.0. Anal. calc. for C_20_H_12_N_4_O_3_: C, 67.41; H, 3.39; N, 15.72. Found: C, 67.66; H, 3.58; N, 15.40.

*(E,Z)-2-(5(6)-Nitrobenzimidazol-2-yl)-3-[5-(4-nitrophenyl)furan-2-yl]acrylonitrile **18***.

Compound **18** was prepared 5-(4-nitrophenyl)furan-2-carbaldehyde **1a** (0.07 g, 0.32 mmol) and 2-cyanomethyl-5(6)-nitrobenzimidazole **16b** (0.07 g, 0.32 mmol) in absolute ethanol (5 mL) after refluxing for 1 h. The resulting product was separated by column chromatography on SiO_2_ using dichloromethane/methanol as eluent to yield 0.05 g (41%) of orange powder; m.p. > 300 °C. **a:** ^1^H NMR (300 MHz, DMSO-*d*_6_) δ = 13.70 (s, 1H, NH_benzim._), 8.41 (s, 1H, Ar-H), 8.33 (d, *J* = 8.9 Hz, 2H, Ar-H), 8.24 (s, 1H, Ar-H), 8.14–8.06 (m, 3H, Ar-H), 7.72 (d, *J* = 8.8 Hz, 1H, Ar-H), 7.65 (d, *J* = 3.9 Hz, 1H, Ar-H), 7.51 (d, *J* = 3.8 Hz, 1H, Ar-H). **b:** ^1^H NMR (300 MHz, DMSO-*d*_6_) δ = 13.70 (s, 1H, NH_benzim._), 8.60 (s, 1H, Ar-H), 8.33 (d, *J* = 8.9 Hz, 2H, Ar-H), 8.14–8.06 (m, 3H, Ar-H), 7.94 (s, 1H, Ar-H), 7.85 (d, *J* = 8.9 Hz, 1H, Ar-H_._), 7.69 (d, *J* = 3.9 Hz, 1H, Ar-H), 7.56 (d, *J* = 3.8 Hz, 1H, Ar-H). ^13^C NMR (75 MHz, DMSO-*d*_6_) δ = 155.6, 150.1, 147.5, 143.6, 134.7, 131.9, 125.8 (2C), 125.0 (2C), 124.4, 116.5, 113.9, 97.6. Anal. calc. for C_20_H_11_N_5_O_5_: C, 59.85; H, 2.76; N, 17.45. Found: C, 59.86; H, 2.78; N, 17.44.

*(E)-2-(Benzothiazol-2-yl)-3-[5-(4-nitrophenyl)furan-2-yl]acrylonitrile **19***.

Compound **19** was prepared 5-(4-nitrophenyl)furan-2-carbaldehyde **1a** (0.13 g, 0.57 mmol) and 2-cyanomethylbenzothiazole **16c** (0.10 g, 0.57 mmol) in absolute ethanol (7 mL) after refluxing for 1 h and recrystallization from ethanol to yield 0.19 g (90%) of orange powder; m.p. 240–242 °C. ^1^H NMR (300 MHz, DMSO-*d*_6_) δ = 8.30 (d, *J* = 8.7 Hz, 2H, Ar-H), 8.22 (s, 1H, Ar-H), 8.12 (d, *J* = 7.9 Hz, 1H, Ar-H), 8.07 (d, *J* = 8.7 Hz, 2H, Ar-H), 8.01 (d, *J* = 8.0 Hz, 1H, Ar-H), 7.62 (d, *J* = 3.7 Hz, 1H, Ar-H), 7.54 (t, *J* = 7.1 Hz, 1H, Ar-H), 7.52 (d, *J* = 3.8 Hz, 1H, Ar-H_._), 7.46 (t, *J* = 7.5 Hz, 1H, Ar-H). ^13^C NMR (75 MHz, DMSO-*d*_6_) δ = 163.1, 155.8, 153.6, 150.2, 147.7, 135.0, 134.8, 132.1, 127.6, 126.6, 126.0 (2C), 125.0 (3C), 123.5, 122.9, 116.8, 113.9, 101.7. Anal. calc. for C_20_H_11_N_3_O_3_S: C, 64.33; H, 2.97; N, 11.25. Found: C, 64.31; H, 2.98; N, 11.24.

*(E)-2-(Benzimidazol-2-yl)-3-[5-(4-chlorophenyl)furan-2-yl]acrylonitrile **20***.

Compound **20** was prepared from 5-(4-chlorophenyl)furan-2-carbaldehyde **1b** (1.24 g, 6.00 mol) and 2-cyanomethylbenzimidazole **16a** (1.00 g, 6.00 mol) in absolute ethanol (20 mL) after refluxing for 2 h. Resulting product was filtered off and recrystallized from ethanol to yield 1.27 g (61%) of orange powder; m.p. 292–294 °C. ^1^H NMR (300 MHz, DMSO-*d*_6_) δ = 13.00 (s, 1H, NH_benzim._), 8.15 (s, 1H, Ar-H), 7.94 (d, 2H, *J* = 8.6 Hz, Ar-H), 7.84 (d, 1H, *J* = 8.8 Hz, Ar-H), 7.63 (d, 2H, *J* = 8.6 Hz, Ar-H), 7.50 (d, 1H, *J* = 8.8 Hz, Ar-H), 7.42 (d, 1H, J = 3.7 Hz, Ar-H), 7.40 (d, 1H, J = 3.7 Hz, Ar-H), 7.24 (brs, 2H, Ar-H). ^13^C NMR (75 MHz, DMSO-*d*_6_) δ = 156.3, 149.1, 148.0, 144.0, 134.2, 130.2, 129.8 (2C), 128.2, 126.7 (2C), 125.7, 123.0, 119.5, 117.0, 112.0, 110.8, 109.6, 97.5. Anal. calc. for C_20_H_12_ClN_3_O: C, 69.47; H, 3.50; N, 12.15. Found: C, 69.20; H, 3.33; N, 12.45.

*(E,Z)-3-[5-(4-Chlorophenyl)furan-2-yl]-2-(5(6)-nitrobenzimidazol-2-yl)acrylonitrile **21***.

Compound **21** was prepared from 5-(4-chlorophenyl)furan-2-carbaldehyde **1b** (0.10 g, 0.48 mmol) and 2-cyanomethyl-5(6)-nitrobenzimidazole **16b** (0.10 g, 0.48 mmol) in absolute ethanol (5 mL) after refluxing for 1 h. Resulting product was separated by column chromatography on SiO_2_ using dichloromethane/methanol as eluent to yield 0.07 g (35%) of orange powder; m.p. > 300 °C. **a:** ^1^H NMR (600 MHz, DMSO-*d*_6_) δ = 13.62 (s, 1H, NH_benzim._), 8.42 (s, 1H, Ar-H), 8.21 (s, 1H, Ar-H), 8.09 (dd, *J* = 8.9, 2.2 Hz, 1H, Ar-H), 7.90 (d, *J* = 8.6 Hz, 2H, Ar-H), 7.72 (d, *J* = 8.8 Hz, 1H, Ar-H), 7.57 (d, *J* = 8.6 Hz, 2H, Ar-H), 7.45 (d, *J* = 3.7 Hz, 1H, Ar-H), 7.41 (d, *J* = 3.7 Hz, 1H, Ar-H). **b:** ^1^H NMR (600 MHz, DMSO-*d*_6_) δ = 13.62 (s, 1H, NH_benzim._), 8.454 (s, 1H, Ar-H), 8.22 (s, 1H, Ar-H), 7.90–7.89 (m, 1H, Ar-H), 7.86 (s, 1H, Ar-H), 7.59–7.57 (m, 1H, Ar-H), 7.39 (d, *J* = 8.6 Hz, 2H, Ar-H), 7.32 (d, *J* = 8.6 Hz, 2H, Ar-H), 7.29 (d, *J* = 3.7 Hz, 1H, Ar-H). ^13^C NMR (75 MHz, DMSO-*d*_6_) δ = 157.1, 148.9, 143.5, 134.5, 132.1, 129.8 (2C), 127.9, 126.8 (2C), 124.7, 116.7, 111.1, 96.1. Anal. calc. for C_20_H_11_ClN_4_O_3_: C, 61.47; H, 2.84; N, 14.34. Found: C, 61.46; H, 2.86; N, 14.32.

*(E)-2-(Benzothiazol-2-yl)-3-[5-(4-chlorophenyl)furan-2-yl]acrylonitrile **22***.

Compound **22** was prepared from 5-(4-chlorophenyl)furan-2-carbaldehyde **1b** (0.12 g, 0.57 mmol) and 2-cyanomethylbenzothiazole **13** (0.10 g, 0.57 mmol) in absolute ethanol (7 mL) after refluxing for 1 h and recrystallization from ethanol to yield 0.17 g (81%) of yellow powder; m.p. 190–192 °C. ^1^H NMR (300 MHz, DMSO-*d*_6_) δ = 8.24 (s, 1H, Ar-H), 8.16 (d, *J* = 8.0 Hz, 1H, Ar-H), 8.05 (d, *J* = 7.9 Hz, 1H, Ar-H), 7.94 (d, *J* = 8.6 Hz, 2H, Ar-H), 7.62 (d, *J* = 8.7 Hz, 2H, Ar-H), 7.58–7.54 (m, 1H, Ar-H), 7.52 (d, *J* = 3.7 Hz, 1H, Ar-H), 7.51–7.46 (m, 1H, Ar-H), 7.44 (d, *J* = 3.7 Hz, 1H, Ar-H). ^13^C NMR (75 MHz, DMSO-*d*_6_) δ = 163.9, 157.7, 154.0, 149.3, 135.2, 135.0, 132.6, 130.3 (2C), 128.4, 128.0, 127.3 (2C), 126.9, 126.1, 123.7, 123.3, 117.4, 111.6, 100.5. Anal. calc. for C_20_H_11_ClN_2_OS: C, 66.21; H, 3.06; N, 7.72. Found: C, 66.20; H, 3.08; N, 7.73.

### 3.2. Pharmacological/Biological Assays

#### 3.2.1. Antitumor Activity in 2D and 3D Cell Culture Assays In Vitro

Test compounds.

Doxorubicin was purchased from Apollo (BID0120; Opelika, AL, USA), staurosporine was purchased from Biotrend (BS0188; Zurich, SUI, Switzerland) and vandetanib was purchased from Selleckchem (S1046, Houston, TX, USA). Test compounds were synthesized by a research group at the Department of Organic Chemistry, Faculty of Chemical Engineering and Technology, University of Zagreb. Mother plates (96-well-V plates, polypropylene, Greiner Bio-one, Cat. 651201) with serial dilutions of compounds in pure DMSO were prepared from 10 mM DMSO stock solutions on Janus automatic pipetting workstation (Perkin-Elmer). Compounds were diluted 1:3. We transferred 500 nL (for 2D cell culture) or 400 nL (for 3D cell culture) of the compound from a mother plate to test plate by using Mosquito (TTP labtech). DMSO percentage in test concentrations was 0.5–1.0%. Starting concentration of the test compounds and standard compound doxorubicin was 50 µM (2D cell culture) or 100 µM (3D cell culture). Starting concentration of staurosporine was 10 µM in both, 2D and 3D cell culture assays, while starting concentration of vandetanib was 25 µM (2D cell culture) or 50 µM (3D cell culture).

Cell cultures.

Human lung cancer cell lines A549 (ATCC CCL-185), HCC827 (ATCC CRL-2868) and NCI-H358 (ATCC CRL-5807) and human lung fibroblast cell line MRC-5 (ATCC CCL-171) were purchased from American Type Culture Collection (ATCC, Manassas, VA, USA). Cells were maintained in appropriate medium, recommended by supplier medium, supplemented with 10% heat inactivated fetal bovine serum and penicillin/streptomycin, in an incubator (INCO2, Memmert, Germany) in a humidified atmosphere of 5% CO_2_ and 95% O_2_ at 37 °C.

High-Throughput 2D and 3D Drug Screening Setup.

2D cell culture assay.

Cells were grown in 96-well cell star polystyrene plates. We seeded 10,000 cells/well in wells 4 h prior to the treatment with different compounds concentrations and incubated for 72 h. A cell viability assay was performed according to the manufacturer’s instructions, using CellTiter 96 aqueous solution, MTS kit (Promega, Madison, WI, USA). After 0.5–2 h of cell incubation with MTS in 2D cell culture, the plates were read using PE EnVision absorbance at 490 nm. The results for each of the tested compounds are reported as growth percentages from two independent concentrations curves compared with the untreated control cells after drug exposure.

3D cell culture assay.

Cells were grown in 96-well Perfecta 3D hanging drop plates 5000 cell/well for 4 days until spheres were formed (and checked under the microscope). After sphere formation cells were treated with compounds followed by 72 h incubation. Cell viability assay was performed according to the manufacturer’s instructions, using CellTiter GLO 3D for 3D cell culture (Promega); cell incubation was 5 min on the shaker followed by 25 min incubation in the dark. Plates were read using PE EnVision luminescence.

The results for each of the tested compounds are reported as growth percentages from two independent concentrations curves compared with the untreated control cells after drug exposure.

BrdU Proliferation Assay.

Proliferation assay was performed using Cell Proliferation ELISA, BrdU (Sigma, St. Louis, MO, USA). Briefly, 48 h hours after compound addition, 10 µM of BrdU was added to each well and incubated for the following 24 h. After incubation, a single cell suspension was made for 3D cell culture in the new 96-well plate. Plates were centrifuged and supernatants were removed from each plate (for 2D and 3D cell culture) followed by 60 min incubation at 60 °C. The rest of the proliferation assay was performed according to the manufacturer’s instructions.

Statistical Analysis.

Calculation of IC_50_ data, curves and QC analysis was performed by using Excel tools and GraphPadPrism software (La Jolla, CA, USA), v. 5.03. In brief, individual concentration–effect curves are generated by plotting the logarithm of the tested concentration of tested compounds (X) versus corresponding percent inhibition values (Y) using least squares (ordinary) fit. Best fit IC_50_ values are calculated using Log(inhibitor) versus normalized response–variable slope equation, where Y = 100/(1 + 10((LogIC_50_–X) × HillSlope)). QC criteria parameters (Z’, S:B, R2, HillSlope) were checked for every IC_50_ curve.

#### 3.2.2. In Vitro Antimicrobial Activity

Materials.

In addition to synthesized compounds, standard antibiotics ampicilin, ceftazidime, ciprofloxacin, gentamycin and voriconazole from USP were tested. Bacterial strains tested were Gram-negative *Escherichia coli* ATCC 25922 strain and Gram-positive *Staphylococcus aureus* ATCC 29213 strain. *Saccharomyces cerevisiae* ATCC 7752 strain was tested as a eukaryotic model organism. Synthesized compounds were prepared as 10 mM DMSO solutions and tested in the final concentration range of 100–0.2 µM. Standard antibiotics were prepared as 5 mg/mL DMSO solutions and tested in the final concentration range of 64–0.125 µg/mL.

Methods.

Broth microdilution testing was performed according to CLSI (Clinical Laboratory Standards Institute) guidelines. MIC (minimal inhibitory concentration) value was defined as the last tested concentration of compound at which there is no visible growth of bacteria. Inoculums for each microorganism were prepared using the direct colony suspension method where broth solutions that achieved turbidity equivalent to 0.5 McFarland standard were additionally diluted 100x with Ca adjusted MH media (Becton Dickinson). All test plates were incubated for 16–24h at 37 °C. MIC values for reference antibiotics against quality control strains were used for confirming the validity of the screen. Clinical and Laboratory Standards Institute (CLSI) guidelines. Methods for dilution antimicrobial susceptibility tests for bacteria that grow aerobically M07, 11th edition, 2018. and Clinical and Laboratory Standards Institute (CLSI) guidelines. Performance standards for antimicrobial susceptibility testing M100, 28th edition, 2018.

#### 3.2.3. DNA Binding Study

All compounds were soluble in water (**5, 8,** and **15** in *c* = 3 × 10^−3^ mol dm^−3^, **6** and **9** in *c* = 1.72 × 10^−3^ mol dm^−3^ and 1.32 × 10^−3^ mol dm^−3^). Due to the small aggregation of **6** and **9** in water, a stock solution was also prepared in DMSO (*c* = 3 × 10^−3^ mol dm^−3^) to check the dependence of UV/Vis changes on concentration increase.

All other experiments with **5**, **6**, **8**, **9** and **15** (*c* = 3 × 10^−3^ mol dm^−3^) were performed by adding small aliquots of aqueous stock solution to buffer solutions (0.05 M Na cacodylate) at 25 °C using appropriate 1cm path quartz cuvettes. Absorption maxima and corresponding molar extinction coefficients (ε) are given in Table S1 and Figures S1 and S2. The UV/vis spectra were recorded on a Varian Cary 100 Bio spectrophotometer, CD spectra on JASCO J815 spectrophotometer and fluorescence spectra on a Varian Cary Eclipse spectrophotometer.

Calf thymus ctDNA, poly(dAdT)_2_ and poly dA-poly dT were purchased in Sigma-Aldrich and dissolved in Na-cacodylate buffer, *I* = 0.05 mol dm^−3^, pH = 7. The ctDNA was additionally sonicated and filtered through a 0.45 mm filter [51]. Polynucleotide concentration was determined spectroscopically as the concentration of phosphates [52]. Spectrophotometric titrations were performed at pH = 7 (*I* = 0.05 mol dm^−3^, sodium cacodylate buffer) by adding portions of polynucleotide solution into the solution of the studied compound for fluorimetric experiments and CD experiments were performed by adding portions of the compound stock solution into the solution of a polynucleotide. In fluorimetric experiments excitation wavelengths of λ_exc_ = 350, 370 and 390 nm were used to avoid the inner filter effect caused due by increasing absorbance of the polynucleotide (Figures S3 and S4). Emission was collected in the range λ_em_ = 400–600 nm (SI). Thermal melting curves for DNA, RNA and their complexes with studied compounds were determined as previously described by following the absorption change at 260 nm as a function of temperature. The absorbance of the ligands was subtracted from every curve and the absorbance scale was normalized. *T*_m_ values are the midpoints of the transition curves determined from the maximum of the first derivative and checked graphically by the tangent method. Every Δ*T*_m_ value here reported was the average of two measurements.

## 4. Conclusions

Within this manuscript, the design, synthesis, structural characterization and biological activity, including antitumor and antibacterial activity in vitro, of novel 2,5-disubstituted furane derivatives bearing benzimidazole or benzothiazole nuclei has been described. The compounds were prepared by using optimized conventional synthetic methods for benzazole derivatives and were substituted with cyano or amidino group on benzazole nuclei as well as with nitro or chloro substituent on furane ring.

Small molecules that target DNA have attracted significant biochemical and biological scientific interest, either by affecting interactions with proteins involved in replication, gene expression and recombination or as spectrophotometric diagnostic tools [53,54,55]. In particular, consecutive AT base pairs participate in DNA bending that can have a functional role in the control of cellular processes [56]. In contrast to ctDNA, the presence of the nitro group or chlorine in selected furane-benzothiazole structures did not influence the binding mode with AT-DNA.

All compounds dominantly bound inside the minor groove of AT-DNA either in form of monomer or dimer and higher-order aggregates. Compound **15** could differentiate between alternating and non-alternating shapes of AT-DNA by induced CD signal.

The results obtained from testing by using 2D and 3D cell culture methods on three human lung cancer cell lines A549, HCC827 and NCI-H358 results revealed compounds with high potential antitumor activity (**5**, **6**, **8**, **9** and **15**). In general, benzothiazole derivatives were more active in comparison to benzimidazole derivatives with exception of benzimidazole **15**.

The same compounds showed also high activity against *S. cerevisiae*; therefore*,* we assume that activity is the consequence of toxicity on eukaryotic cells, which is not an uncommon finding for potential oncology drugs.

However, according to antiproliferative effects, compounds **5, 6, 8** and **9** have a high potential to stop the proliferation of cells and in this way provide antitumor activity, which is a common mode of action of many chemotherapeutics. Additionally, some active compounds (**5** and **9**) showed a shift of activity in 3D cytotoxicity and antiproliferative assay from very active to mildly active compounds, which means that in a natural condition such compounds could be less toxic but still effective against tumor growth and therefore they could be promising new antitumor drugs.

## Figures and Tables

**Table 1:** Cytotoxicity assays on tested compounds in 2D and 3D assays format.

Cytotoxicity Assay IC_50_ Values (µM)							
Comp.	IC_50_ (µM) ± SD; *n* = 2						
	A549		HCC827		NCI-H358		MRC-5
	2D	3D	2D	3D	2D	3D	2D
4	>50	>100	>50	>100	>50	>100	>50
5	8.76 ± 1.69	26.44 ± 13.37	19.33 ± 1.72	43.59 ± 6.45	9.97 ± 0.59	14.80 ± 0.57	21.65 ± 0.51
6	2.12 ± 0.21	4.01 ± 0.95	5.13 ± 0.97	7.02 ± 3.25	0.85 ± 0.05	1.73 ± 0.01	3.11 ± 0.26
7	>50	>100	>50	>100	>50	>100	>50
8	6.74 ± 0.19	9.31 ± 0.78	6.26 ± 0.33	20.46 ± 8.63	6.48 ± 0.11	16.00 ± 9.38	9.88 ± 2.38
9	4.39 ± 0.79	16.81 ± 2.61	5.9 ± 0.58	19.99 ± 7.92	4.48 ± 0.30	15.49 ± 0.07	6.65 ± 0.18
10	>50	>100	>50	>100	>50	>100	>50
11	>50	>100	>50	>100	>50	>100	>50
12	>50	>100	>50	>100	>50	>100	>50
13	>50	>100	>50	>100	>50	>100	>50
14	>50	>100	>50	>100	31.07 ± 0.67	>100	>50
15	24.29 ± 0.40	55.84 ± 8.29	22.70 ± 0.04	65.42 ± 5.04	19.39 ± 0.57	81.62 ± 7.71	>50
17	>50	>100	>50	>100	>50	>100	>50
18	>50	>100	>50	>100	3.50 ± 1.14	>100	>50
19	>50	>100	>50	>100	>50	>100	>50
20	>50	>100	43.53 ± 8.17	9.48 ± 1.15	31.08 ± 4.42	>100	>50
21	>50	>100	>50	>100	3.58 ± 0.95	>100	>50
22	>50	>100	>50	>100	>50	>100	>50
Doxorubicin	1.31 ± 0.11	5.86 ± 1.10	0.32 ± 0.03	1.47 ± 0.12	0.15 ± 0.06	0.99 ± 0.15	0.60 ± 0.01
Staurosporine	0.047 ± 0.007	0.095 ± 0.01	0.033 ± 0.002	0.002 ± 0.0001	0.041 ± 0.007	0.002 ± 0.001	0.031 ± 0.011
Vandetanib	>25	>50	0.58 ± 0.03	0.16 ± 0.04	3.12 ± 35.99	0.38 ± 0.20	11.12 ± 0.00

**Table 2:** Antiproliferation activity on 2,5-furane derivatives in 2D and 3D cell cultures.

Proliferation Assay IC_50_ Values (µM)						
Cpd.	IC_50_ (µM) ± SD; *n* = 2					
	A549		HCC827		NCI-H358	
	2D	3D	2D	3D	2D	3D
4	>50	>100	>50	>100	>50	>100
5	4.76 ± 1.03	65.93 ± 3.66	13.66 ± 3.13	14.69 ± 13.36	3.56 ± 0.18	14.29 ± 0.84
6	2.27 ± 0.13	5.52 ± 0.10	16.69 ± 9.39	7.63 ± 5.00	1.10 ± 0.001	2.36 ± 1.17
7	>50	>100	>50	>100	>50	>100
8	8.78 ± 3.62	19.94 ± 2.19	13.48 ± 0.81	17.75 ± 4.24	6.68 ± 0.15	11.27 ± 0.49
9	9.97 ± 5.37	17.01 ± 3.53	8.69 ± 2.79	20.28 ± 13.87	5.57 ± 0.81	6.39 ± 3.29
10	>50	>100	>50	>100	>50	>100
11	>50	>100	>50	>100	>50	>100
12	>50	>100	>50	16.85 ± 8.33	>50	>100
13	22.73 ± 0.54	>100	>50	>100	15.67 ± 0.86	>100
14	>50	>100	>50	>100	20.20 ± 0.71	>100
15	>50	>100	>50	>100	20.08 ± 2.82	>100
17	>50	>100	>50	>100	>50	>100
18	>50	>100	>50	>100	>50	>100
19	>50	>100	>50	>100	>50	>100
20	43.86 ± 54.27	>100	>50	0.16 ± 0.11	14.36 ± 6.46	>100
21	>50	>100	>50	>100	>50	>100
22	>50	>100	>50	>100	>50	>100
Doxorubicin	0.13 ± 0.01	0.48 ± 0.03	0.25 ± 0.28	1.89 ± 2.30	0.07 ± 0.04	2.30 ± 1.91
Staurosporine	0.035 ± 0.017	0.11 ± 0.001	0.004 ± 0.002	0.003 ± 0.002	0.0005 ± 0.00004	0.02 ± 0.01
Vandetanib	>25	>50	0.20 ± 0.11	0.08 ± 0.02	>25	0.38 ± 0.17

**Table 3:** Antibacterial activity of prepared 2,5-disubstituted furanes.

Comp.	*E. coli* ATCC 25922	*S. aureus* ATCC 29213	*S. cerevisiae* ATCC 7752
	MIC (μM)		
4	>100	>100	>100
5	>100	50	12.5
6	>100	>100	1.6
7	>100	>100	>100
8	>100	12.5	6.25
9	>100	25	3.13
10	>100	>100	>100
11	>100	100	>100
12	>100	>100	100
13	>100	>100	>100
14	50	12.5	50
15	25	6.25	6.25
17	>100	>100	>100
18	>100	>100	>100
19	>100	>100	>100
20	>100	>100	>100
21	>100	>100	>100
22	>100	>100	>100
Ampicillin	4	2	>64
Ceftazidime	<0.125	16	>64
Ciprofloxacin	<0.125	0.25	>64
Gentamycin	0.5	0.25	>64
Voriconazole	>64	>64	<0.125

**Table 4:** The ^a^ Δ*T*_m_ values (°C) of studied ds- polynucleotides upon addition of ratio ^b^ ***r*** = 0.3 of **5, 6, 8, 9,** and **15** at pH 7.0 (sodium cacodylate buffer, *I* = 0.05 mol dm^−3^).

^b^*r* = 0.3	5	6	8	9	15
ctDNA	2.5	3.2	1.7	1.8	0.9
poly (dAdT)_2_	4.8	3.6	3.6	4.6	6.2
Poly dA-poly dT	5.5	5.6	4.3	15.2	11.7

^a^ Error in ΔT_m_: ±0.5 °C; ^b^ ***r** =* [compound]/[polynucleotide].

## Supplementary Materials

The following are available online. Figure S1–S4: spectroscopic characterization of compounds; Figure S5: fluorimetric titrations; Figures S6 and S7: thermal melting experiments; Figure S8: CD experiments; Table S1: electronic absorption data; Table S2: binding constants.

## Sample Availability

Not available.
