# Supplementary material for: Biological Activity of Newly Synthesized Benzimidazole and Benzothizole 2,5-Disubstituted Furane Derivatives
Source: Molecules. 2021 Aug 14;26(16):4935. doi: 10.3390/molecules26164935 (PMC8401404; doi:10.3390/molecules26164935)
Supplement: Supplementary file 1 [file molecules-26-04935-s001.zip › molecules-1319722-supplementary.pdf]

Supporting information

Biological activity of newly synthesized benzimidazole and benzothiazole 2,5-disubstituted furane derivatives

Livio Racané[1‡], Ivo Zlatar[2‡], Nataša Perin[3], Maja Cindrić[3], Vedrana Radovanović[2], Mihailo Banjanac[2], Suresh Shanmugam[5], Marijana Radić Stojković[5], Karmen Brajša[2*], Marijana Hranjec[4*]

1 Department of Applied Chemistry, Faculty of Textile Technology, University of Zagreb, Prilaz baruna Filipovića 28a, 10000 Zagreb, Croatia
2 Pharmacology in vitro, Fidelta Ltd. Prilaz baruna Filipovića 29, Zagreb, Croatia
3 DMPK, Fidelta Ltd. Prilaz baruna Filipovića 29, Zagreb, Croatia
4 Department of Organic Chemistry, Faculty of Chemical Engineering and Technology, University of Zagreb, Marulićev trg 19, 10000 Zagreb, Croatia
5 Ruđer Bošković Institute, Division of Organic Chemistry and Biochemistry, Bijenička cesta 54, 10 000 Zagreb, Croatia

Contents

1. Spectroscopic characterization of benzimidazole and benzothizole 2,5-disubstituted furane derivatives in aqueous solutions.
2. Interactions of benzimidazole and benzothizole 2,5-disubstituted furane derivatives with ds-polynucleotides in neutral medium (pH=7.0)
2.1. Fluorimetric titrations
2.2. Thermal melting experiments
2.3. Circular dichroism (CD) titrations
3. NMR spectra

1. Spectroscopic characterization of benzimidazole and benzothizole 2,5-disubstituted furane derivatives in aqueous solutions

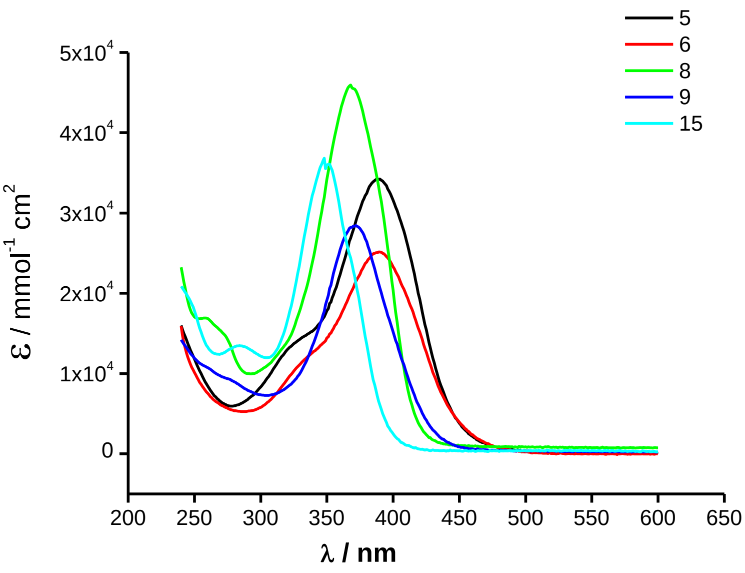

Figure S1. UV/Vis spectra of **5, 6, 8, 9** and **15** at c = 2 × 10^{-5} mol dm^{-3}; pH=7, sodium cacodylate/HCl buffer, I = 0, 05 mol dm^{-3}.

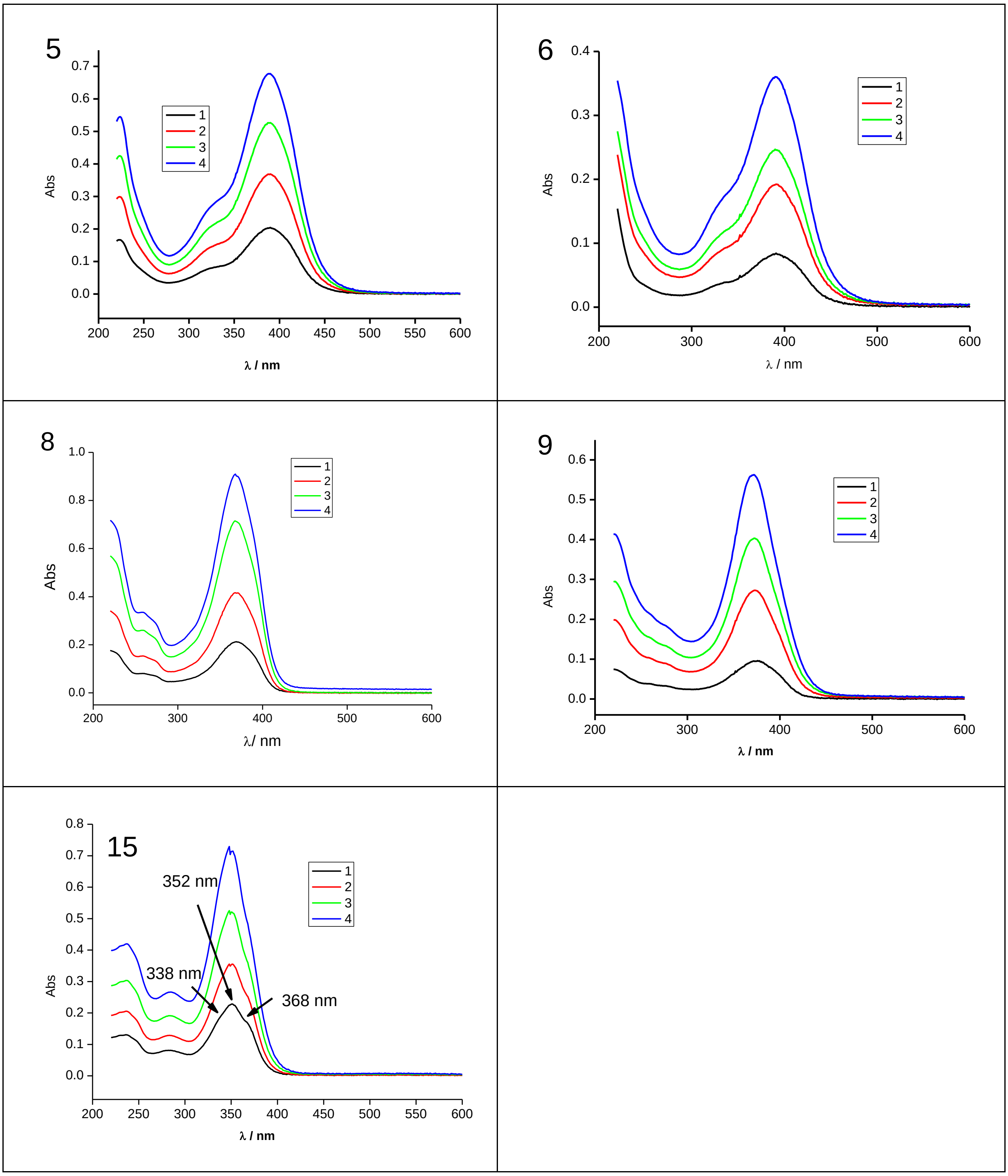

Figure S2. UV/Vis spectra changes of **5, 6, 8, 9** and **15** at different concentrations (concentration range from 5×10^{-6} to 2×10^{-5} mol dm^{-3}) at pH=7, sodium cacodylate buffer, I=0,05 M.

Table S1. Electronic absorption data of **5**, **6**, **8**, **9** and **15**.

	pH = 7,0[a]	
	λ_{max} / nm	$\varepsilon \times 10^3$ / mmol^{-1} cm^2
**5**	390	31.9
**6**	391	17.9
**8**	370	47.8
**9**	373	30.8
**15**	350	32.8

[a] Sodium cacodylate buffer, I = 0,05 mol dm^{-3}, pH = 7,0.

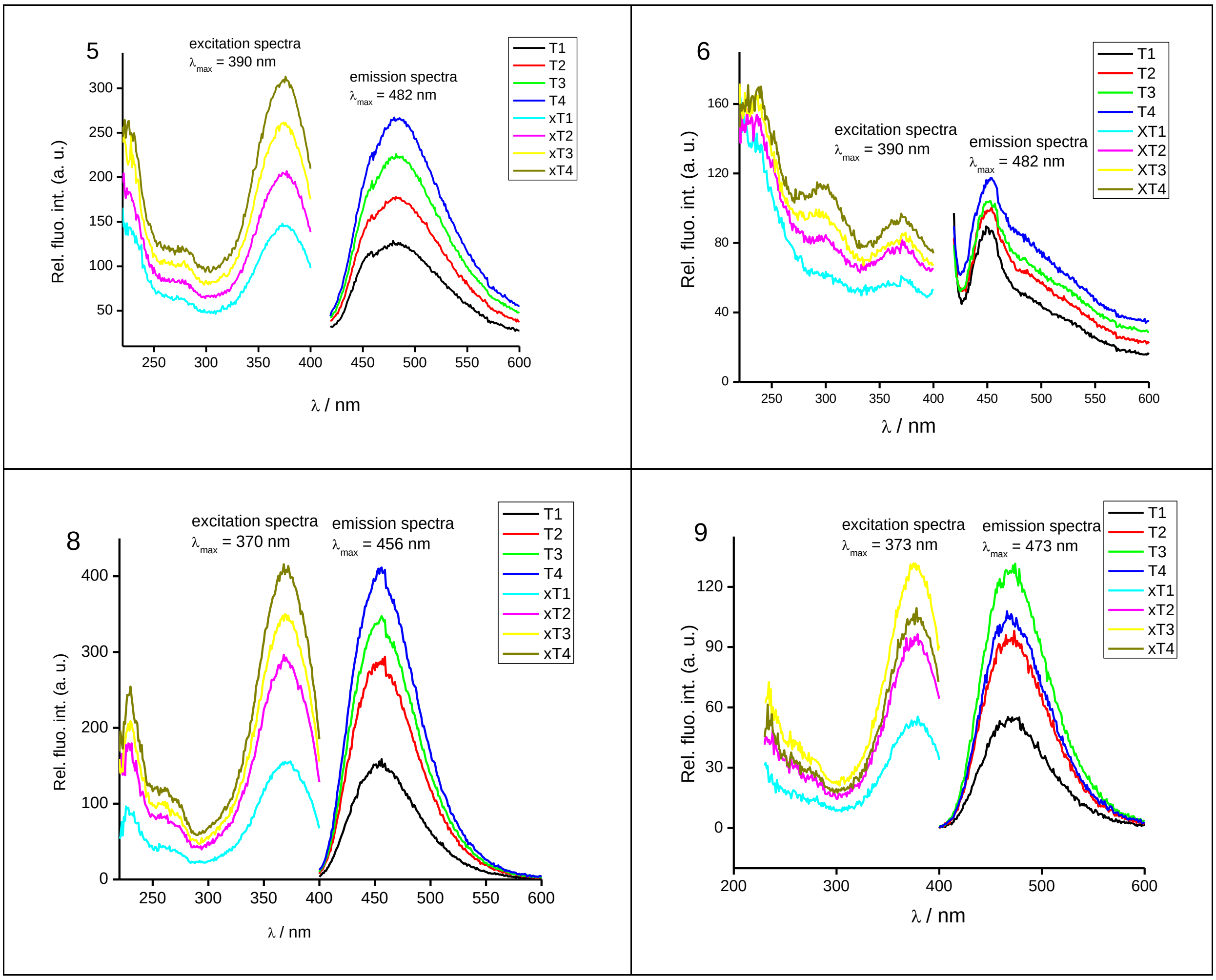

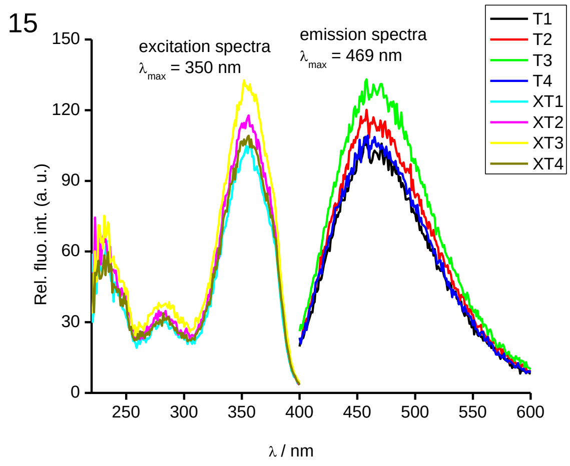

Figure S3. Emission and excitation spectra changes of compounds at different concentrations (concentration range from 5 × 10^{-7} to 2 × 10^{-6} mol dm^{-3}) at pH=7, Na cacodylate buffer, I=0,05 mol dm^{-3}.

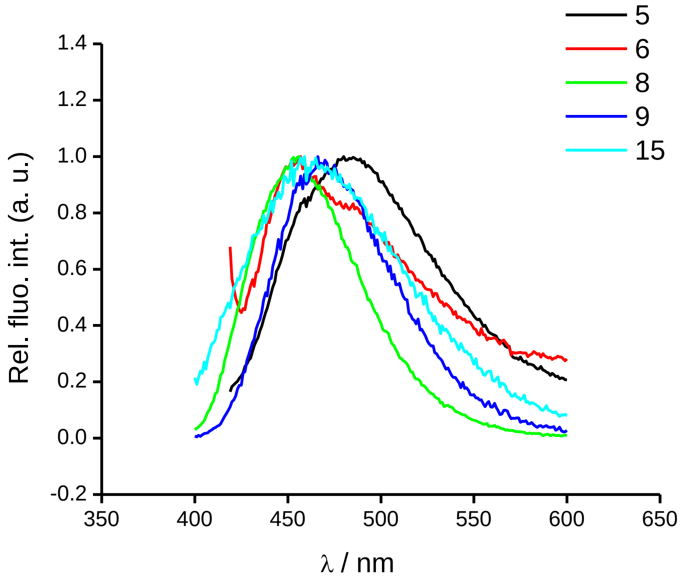

Figure S4. Emission spectra of **5** (λ_{exc}=390nm), **6** (λ_{exc}=390nm), **8** (λ_{exc}=370nm), **9** (λ_{exc}=373nm) and **15** (λ_{exc}=350nm) at concentration, c=2 × 10^{-6} mol dm^{-3}; at pH=7, Na cacodylate buffer, I=0.05 mol dm^{-3}.

2. Interactions of of benzimidazole and benzothizole 2,5-disubstituted furane derivatives with ds-polynucleotides in neutral medium (pH=7.0)

2.1. Fluorimetric titrations

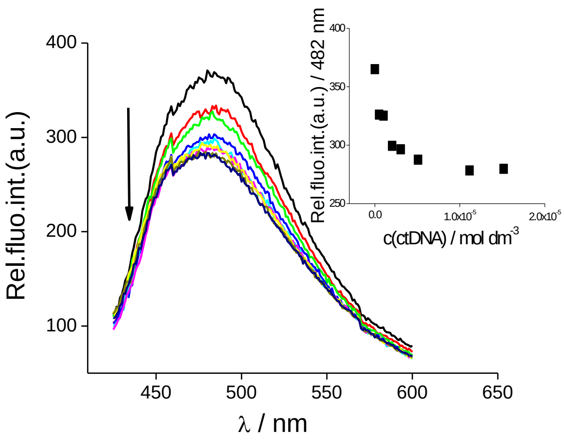

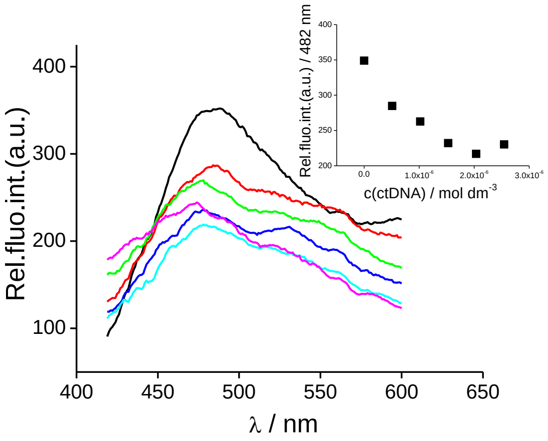

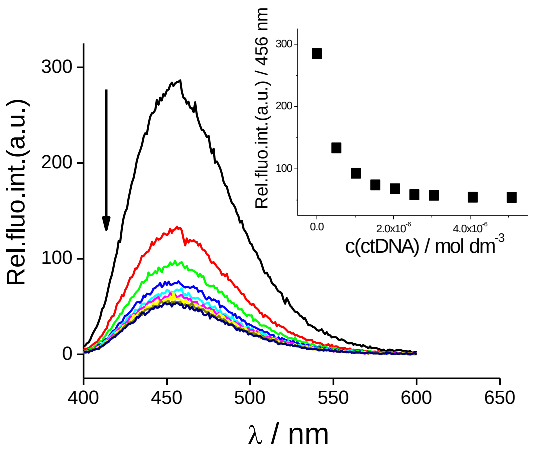

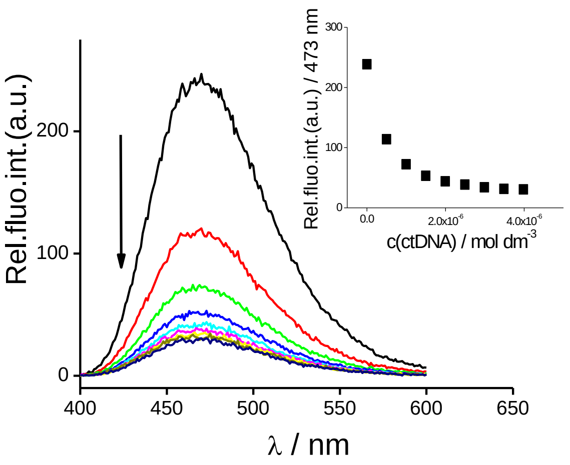

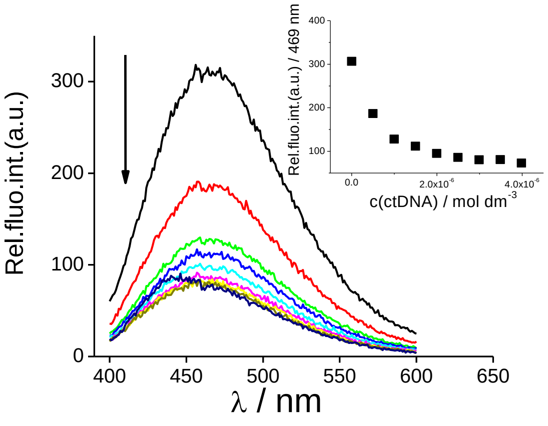

Figure S5. **a)** Changes in fluorescence spectrum of **5** (c= 2.0 × 10^{-6} mol dm^{-3}, λ_{exc}=390 nm) upon titration with ctDNA (c= 5.0 × 10^{-7} – 1.5× 10^{-5} mol dm^{-3}); Inset: Dependence of **5** absorbance at λ_{max} = 482 nm on c(ctDNA); **b)** Changes in fluorescence spectrum of **6** (c= 2.0 × 10^{-6} mol dm^{-3}, λ_{exc}=390 nm) upon titration with ctDNA (c= 5,0 × 10^{-7} –3.5× 10^{-6} mol dm^{-3}); Inset: Dependence of **6** absorbance at λ_{max} = 482 nm on c(ctDNA); **c)** Changes in fluorescence spectrum of **8** (c= 2.0 × 10^{-6} mol dm^{-3}, λ_{exc}=370 nm) upon titration with ctDNA (c= 5,0 × 10^{-7}- 5× 10^{-6} mol dm^{-3}); Inset: Dependence of **8** absorbance at λ_{max} = 456 nm on c(ctDNA); **d)** Changes in fluorescence spectrum of **9** (c= 2.0 × 10^{-6} mol dm^{-3}, λ_{exc}=373nm) upon titration with ctDNA (c= 5.0 × 10^{-6} – 4.0× 10^{-6} mol dm^{-3}); Inset: Dependence of **9** absorbance at λ_{max} = 473 nm on c(ctDNA); **e)** Changes in fluorescence spectrum of **15** (c= 2.0 × 10^{-6} mol dm^{-3}, λ_{exc}=350nm) upon titration with ctDNA (c= 5.0 × 10^{-6} – 4.0× 10^{-6} mol dm^{-3}); Inset: Dependence of **15** absorbance at λ_{max} = 469 nm on c(ctDNA) at pH=7, sodium cacodylate buffer, I = 0,05 mol dm^{-3}.

Table S2. Binding constants (logK_s)[a] and fluorescence intensity ratios[b] calculated from the fluorescence titrations of **5, 6, 8, 9** and **15** with ctDNA at pH = 7,0 (buffer sodium cacodylate, I = 0,05 mol dm^{-3}).

compound	**5**	**6**	**8**	**9**	**15**
logK_s	6.4	[c]	6.3	6.1	6.2
I/I_0[b]	0.8	[c]	0.2	0.5	0.2

[a]Titration data were processed using Scatchard equation, correlation coefficients were >0.97-0.99 for all calculated Ks

[b] I_0 – starting fluorescence intensity of compound, I – fluorescence intensity of **5, 6, 8, 9** and **15** / polynucleotide complex calculated by Scatchard equation.

[c] small / linear fluorescence change or precipitation disabled calculation of stability constant.

2.2. Thermal melting experiments

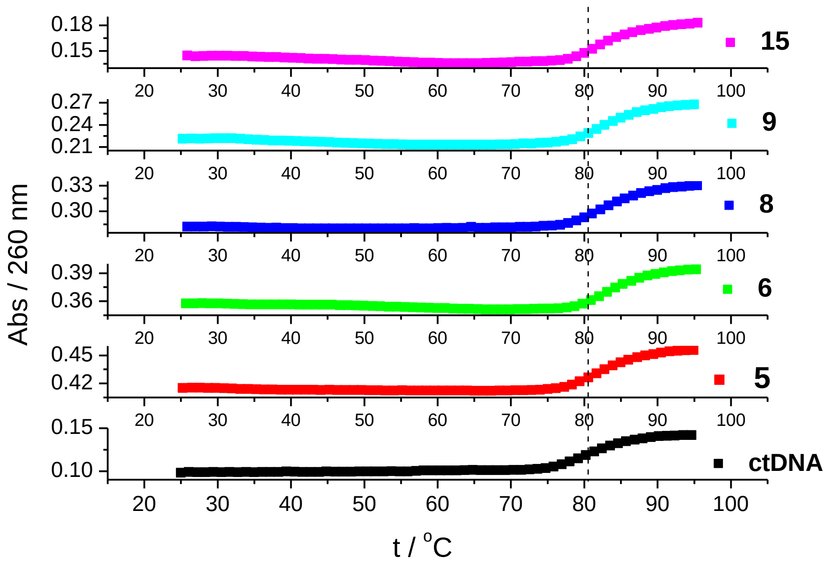

Figure S6. a) Melting curve of **ctDNA** upon addition of ratio, ***r*** ([compound/ [polynucleotide])=0.3 of **5, 6, 8, 9** and **15** at pH = 7.0 (buffer sodium cacodylate, *I* = 0.05 mol dm^{-3}).

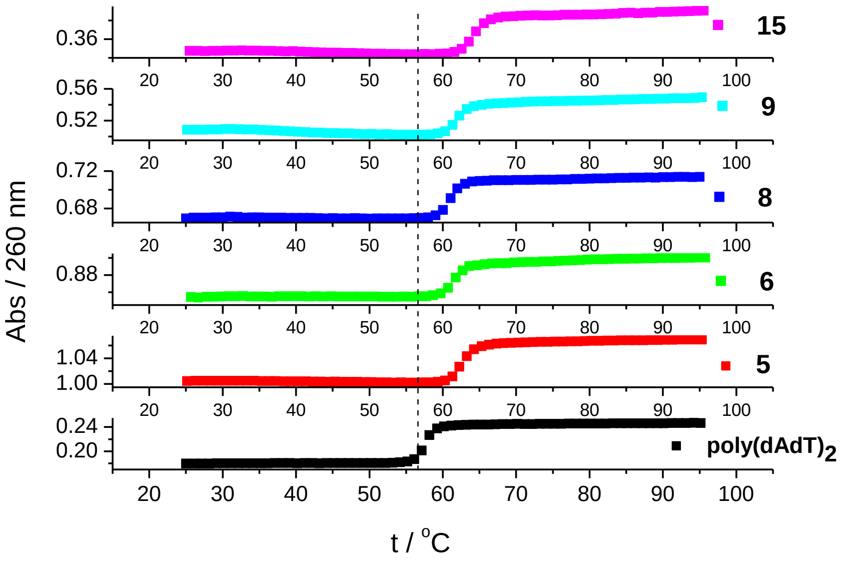

Figure S7. a) Melting curve of **poly(dAdT)2** upon addition of ratio, ***r*** ([compound/ [polynucleotide])=0.3 of **5, 6, 8, 9** and**15** at pH = 7.0 (buffer sodium cacodylate, *I* = 0.05 mol dm^{-3}).

2.3. *Circular dichroism (CD) experiments*

	5	6	8	9	15
	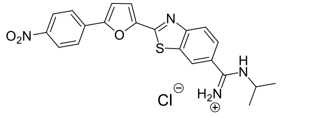	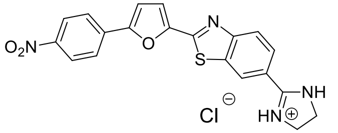	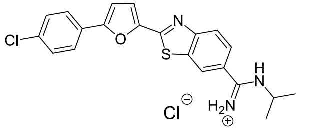	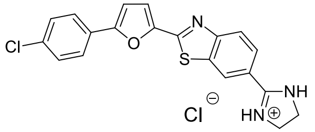	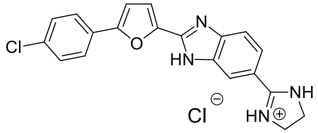
UV	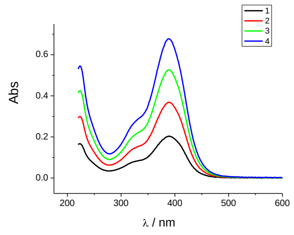	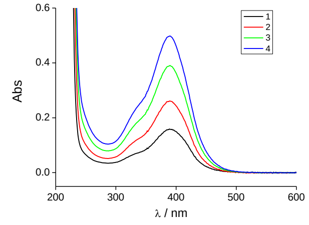	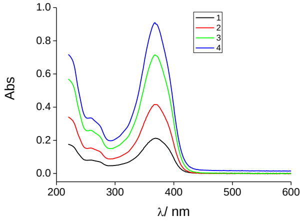	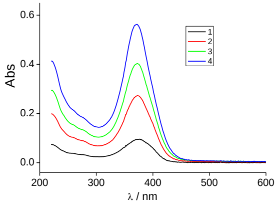	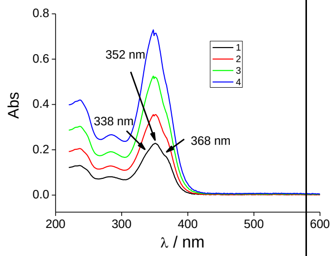
ctDNA	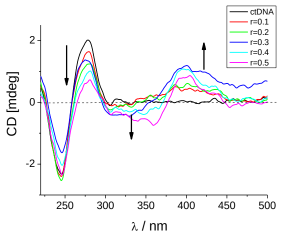	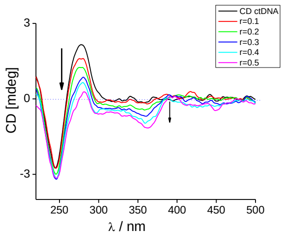	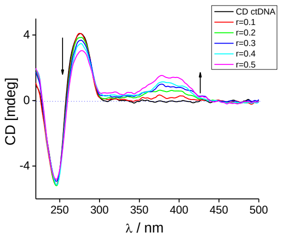	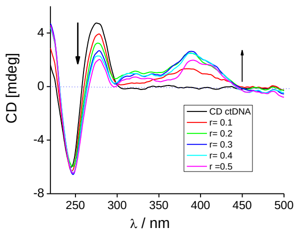	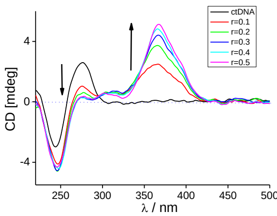

Poly(dAdT)$_2$

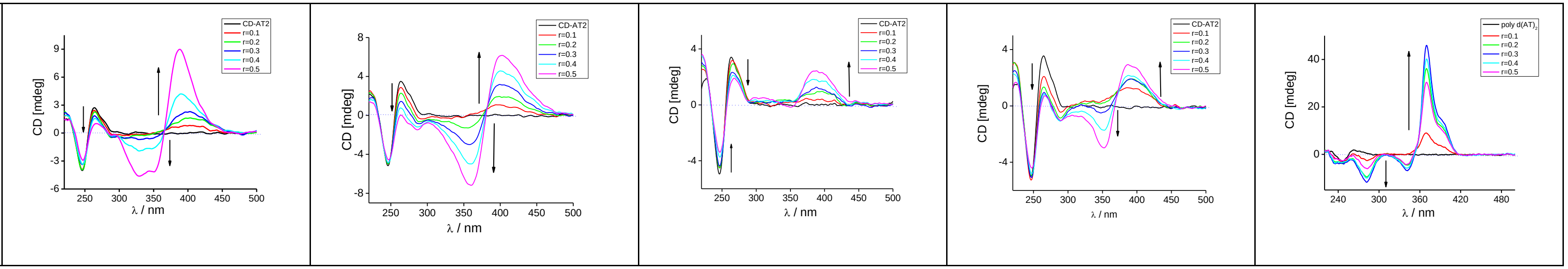

Figure S8. CD titration of ctDNA (c = 3.0 × 10^{-5} mol dm^{-3}) and poly(dAdT)$_2$ (c = 3.0 × 10^{-5} mol dm^{-3}) with **5, 6, 8, 9** and **15** at molar ratios $\boldsymbol{r}$ = [compound] / [polynucleotide] (pH = 7.0, buffer sodium cacodylate, I = 0.05 mol dm^{-3}).

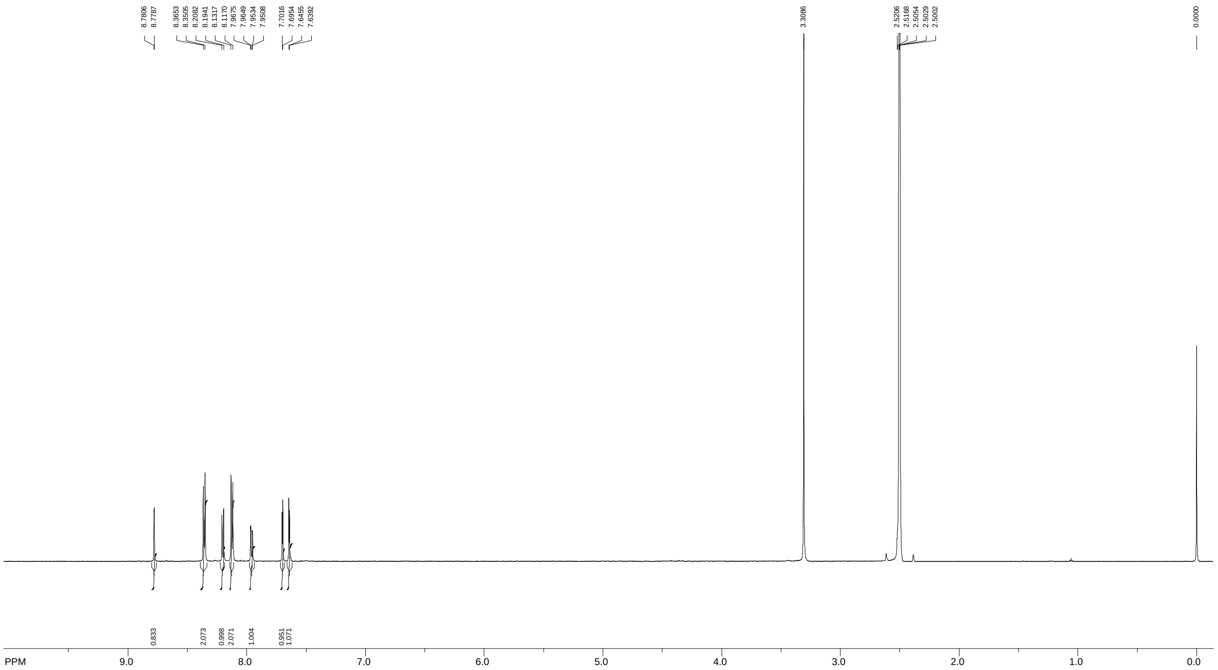

Figure S9. ^{1}H NMR spectrum (DMSO-d_6, 600 MHz) of 6-cyano-2-[5-(4-nitrophenyl)furan-2-yl]benzothiazole **4**

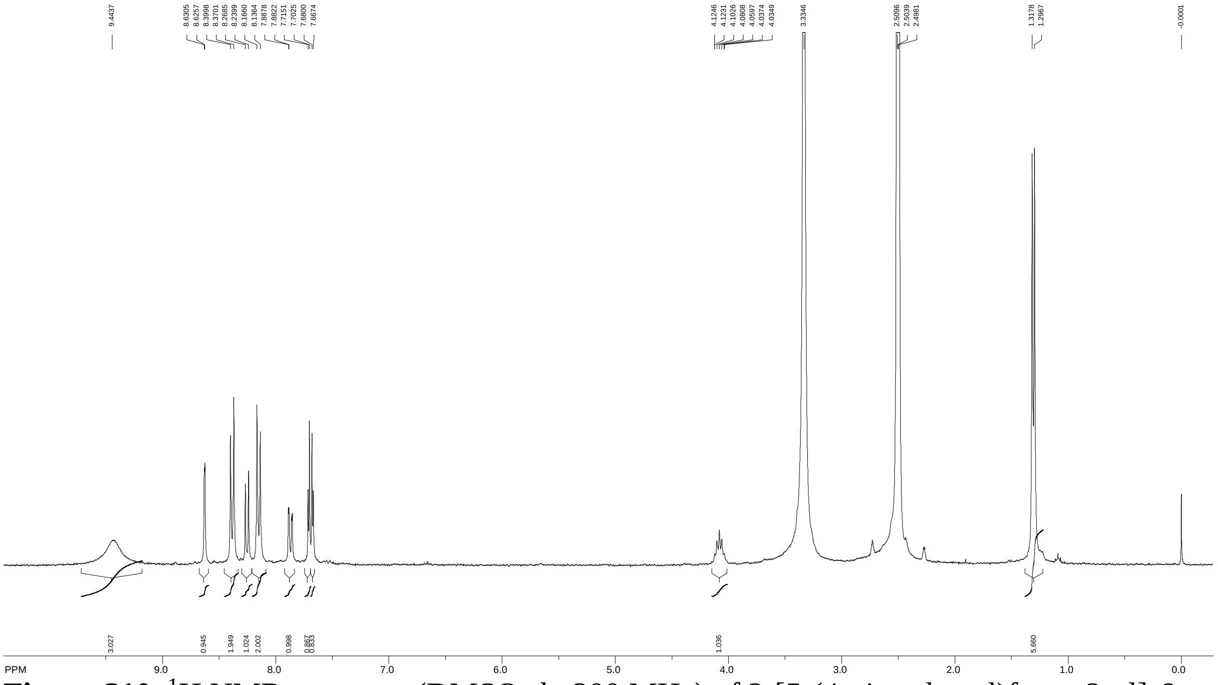

Figure S10. ^{1}H NMR spectrum (DMSO-d_6, 300 MHz) of 2-[5-(4-nitrophenyl)furan-2-yl]-6-*N*-isopropilamidinobenzothiazole hydrochloride **5**

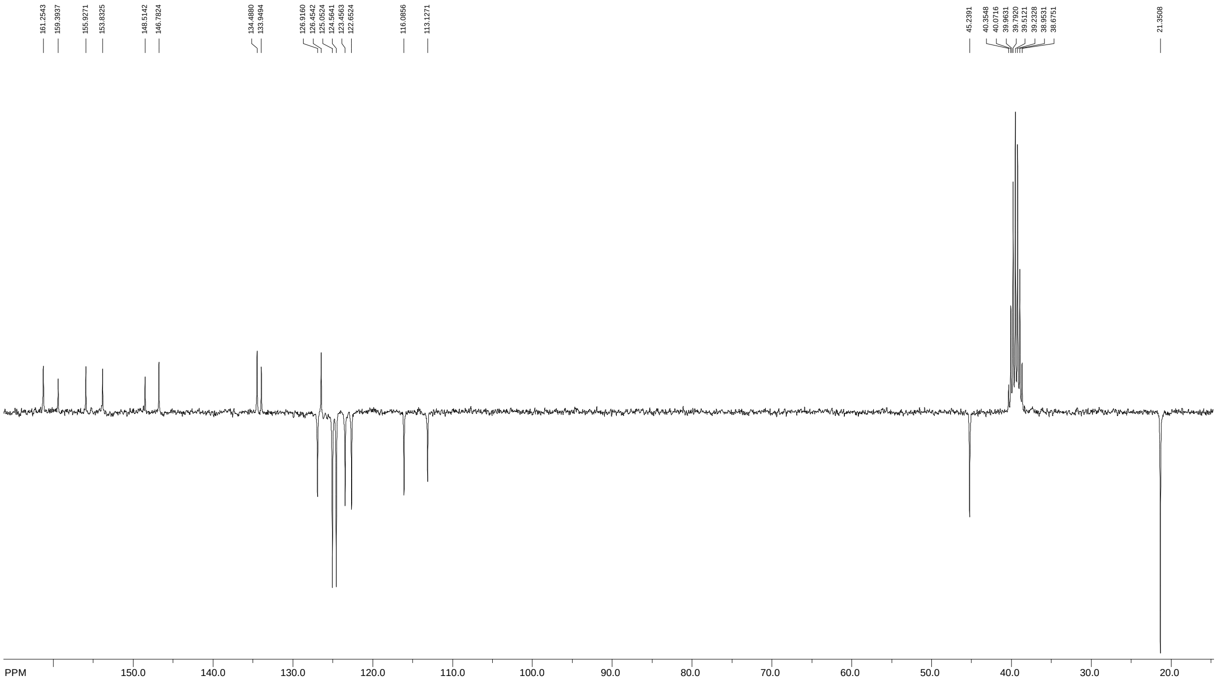

Figure S11. ^{13}C NMR spectrum (DMSO-d_6, 75 MHz) of 2-[5-(4-nitrophenyl)furan-2-yl]-6-*N*-isopropilamidinobenzothiazole hydrochloride **5**

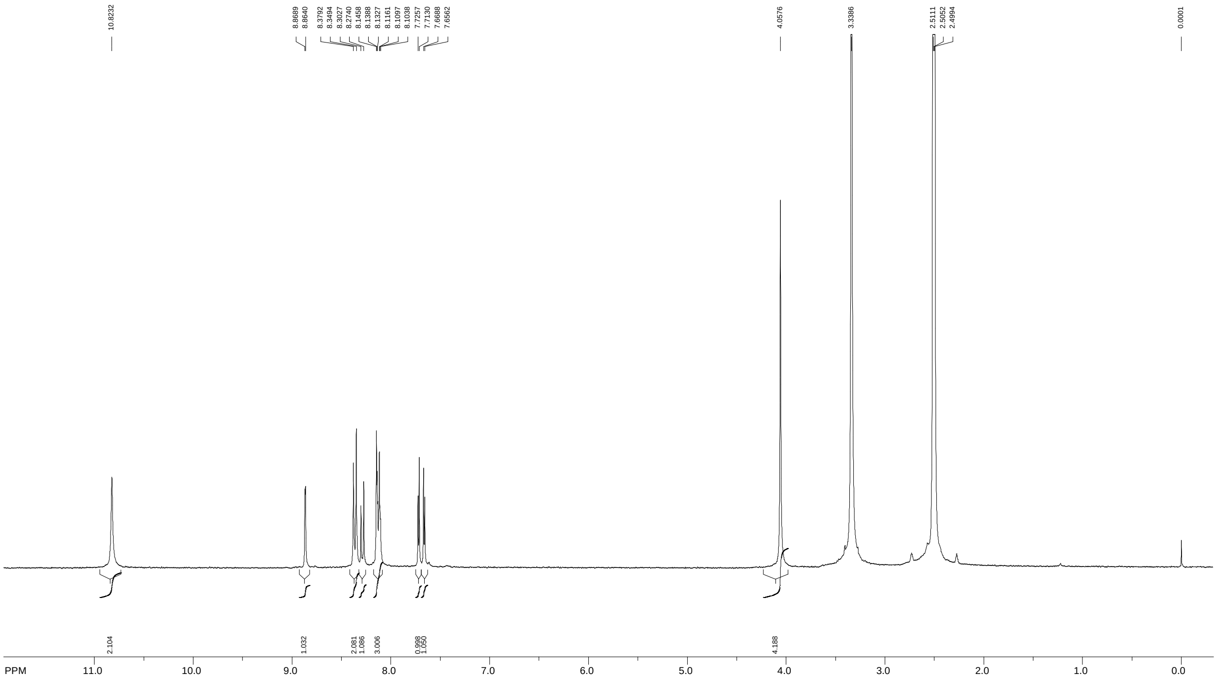

Figure S12. ^{1}H NMR spectrum (DMSO-d_6, 300 MHz) of 6-(4,5-dihydro-1*H*-imidazol-2-yl)-2-[5-(4-nitrophenyl)furan-2-yl]benzothiazole hydrochloride **6**

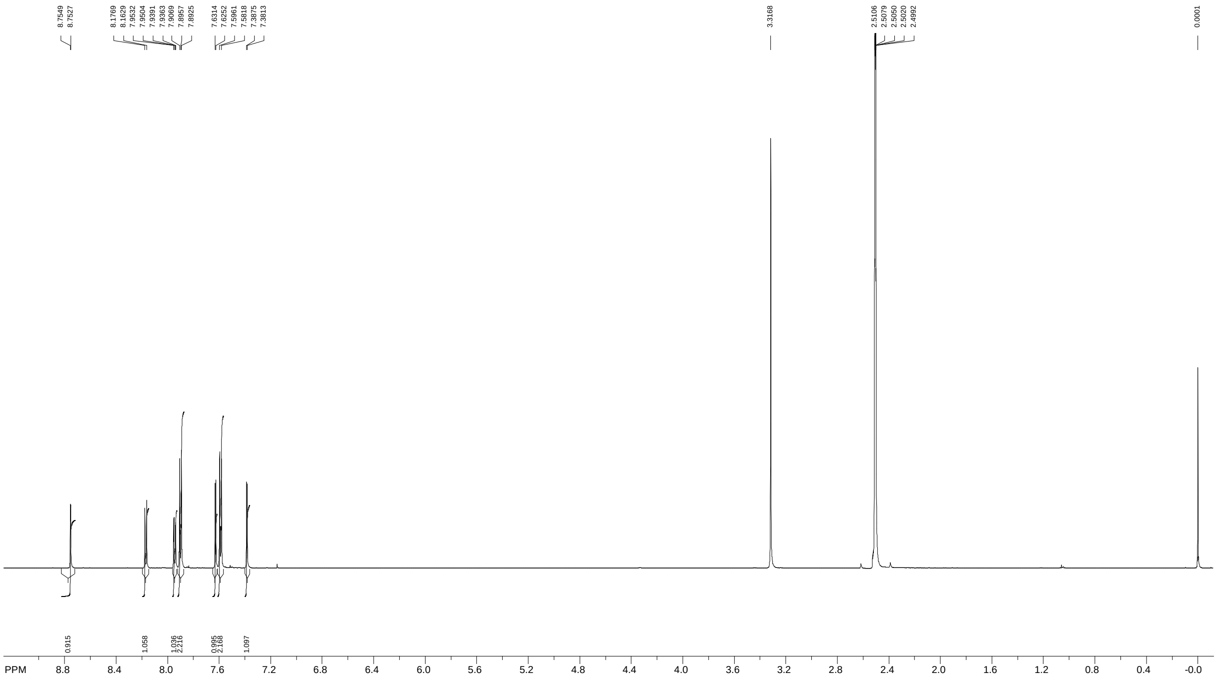

Figure S13. ^{1}H NMR spectrum (DMSO-d_6, 600 MHz) of 2-[5-(4-chlorophenyl)furan-2-yl]-6-cyanobenzothiazole **7**

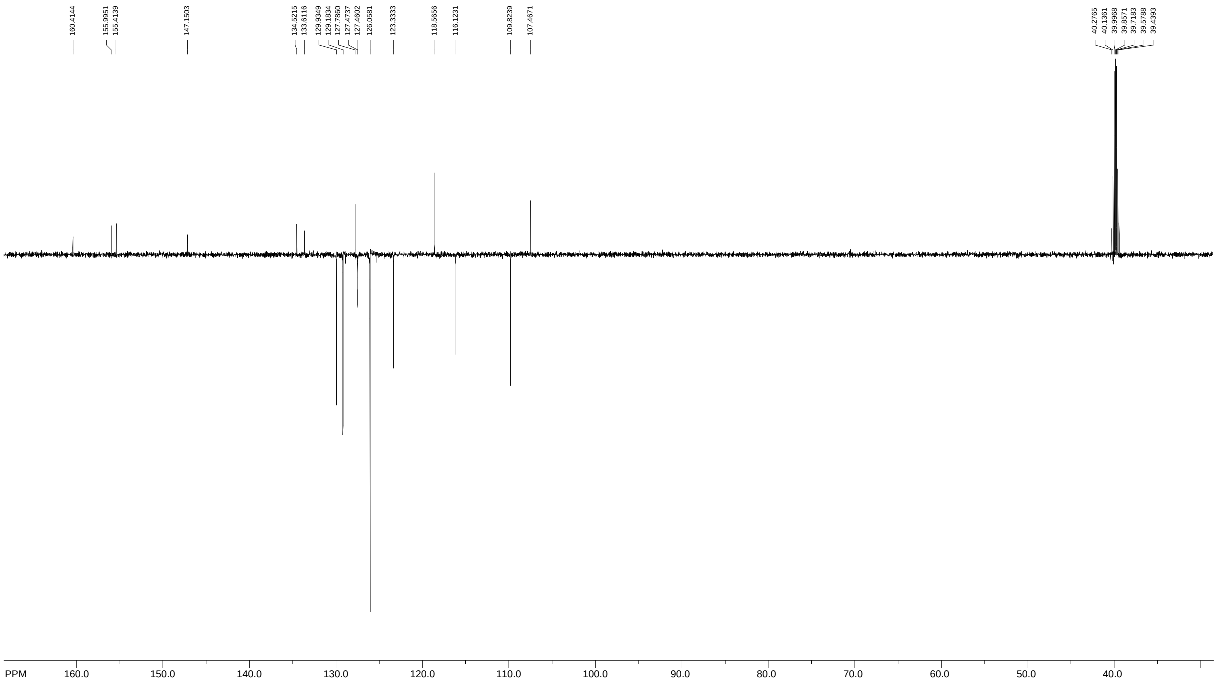

Figure S14. ^{13}C NMR spectrum (DMSO-d_6, 150 MHz) of 2-[5-(4-chlorophenyl)furan-2-yl]-6-cyanobenzothiazole **7**

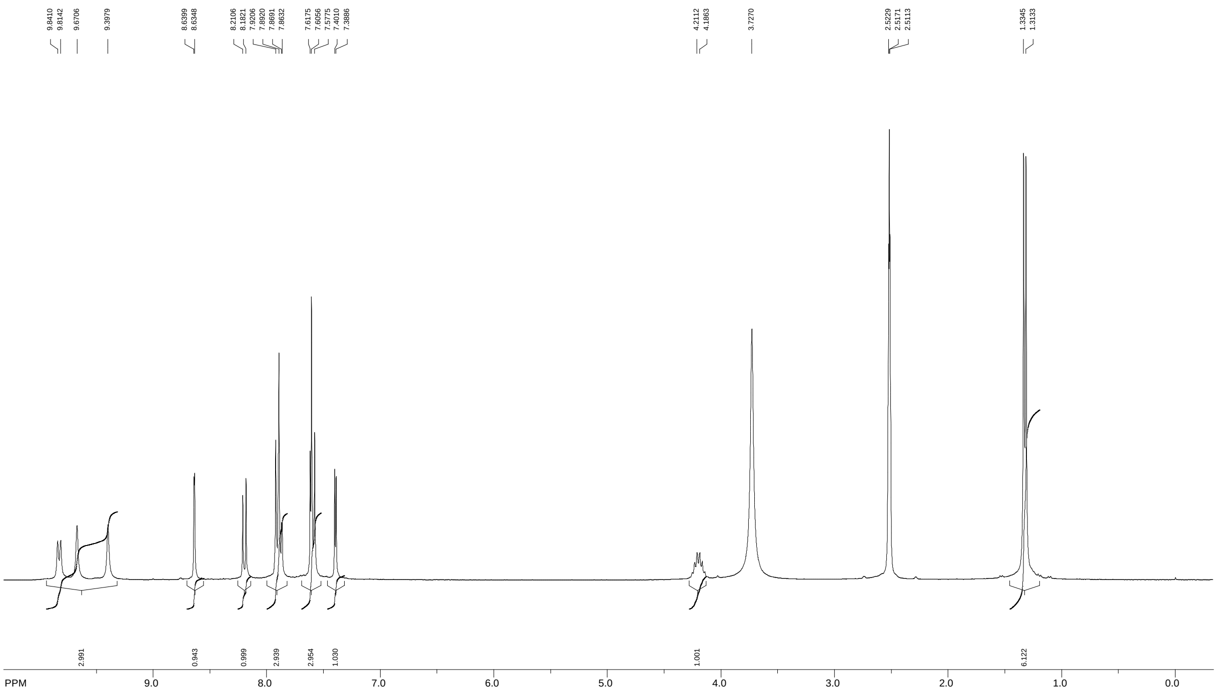

Figure S15. ^{1}H NMR spectrum (DMSO-d_6, 300 MHz) of 2-[5-(4-chlorophenyl)furan-2-yl]-6-*N*-isopropilamidinobenzothiazole hydrochloride **8**

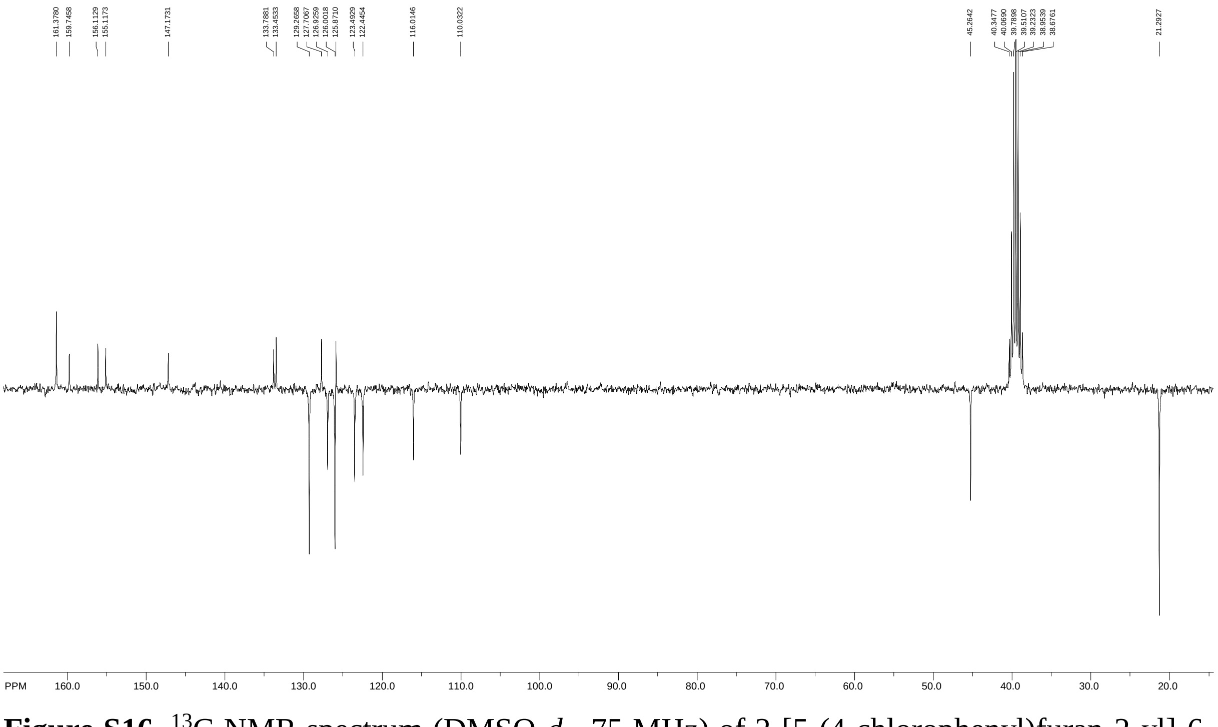

Figure S16. ^{13}C NMR spectrum (DMSO-d_6, 75 MHz) of 2-[5-(4-chlorophenyl)furan-2-yl]-6-*N*-isopropilamidinobenzothiazole hydrochloride **8**

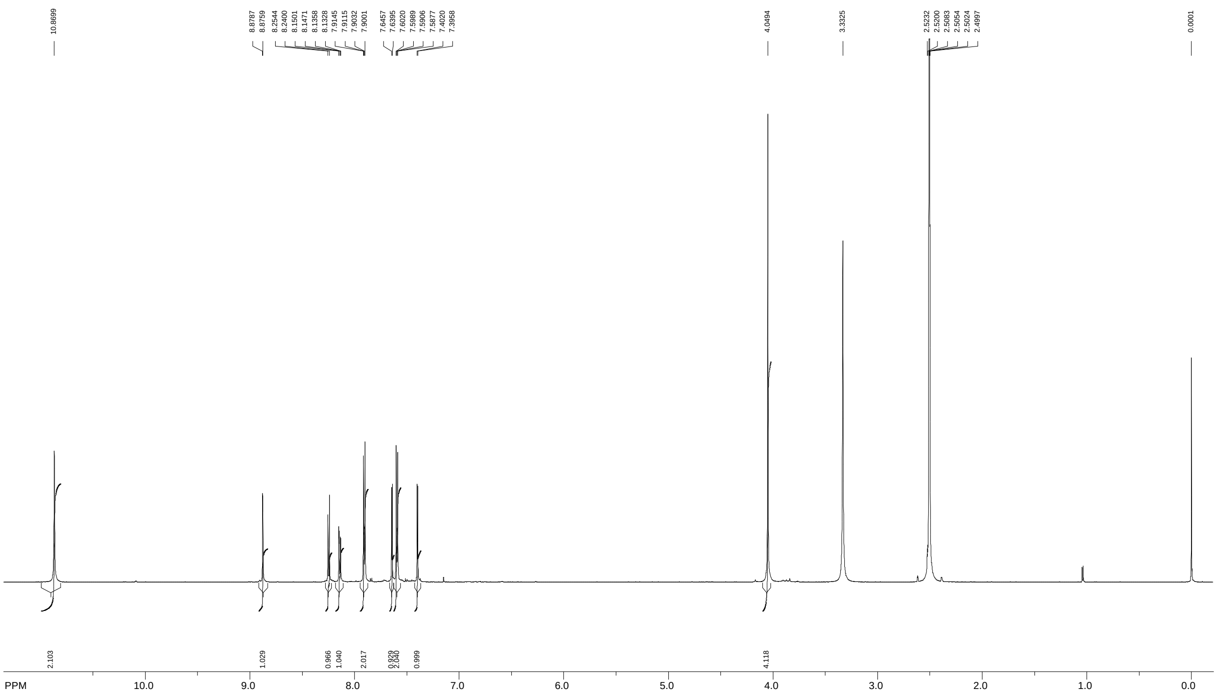

Figure S17. ^{1}H NMR spectrum (DMSO-d_6, 600 MHz) of 2-[5-(4-chlorophenyl)furan-2-yl]-6-(4,5-Dihydro-1*H*-imidazol-2-yl)benzothiazole hydrochloride **9**

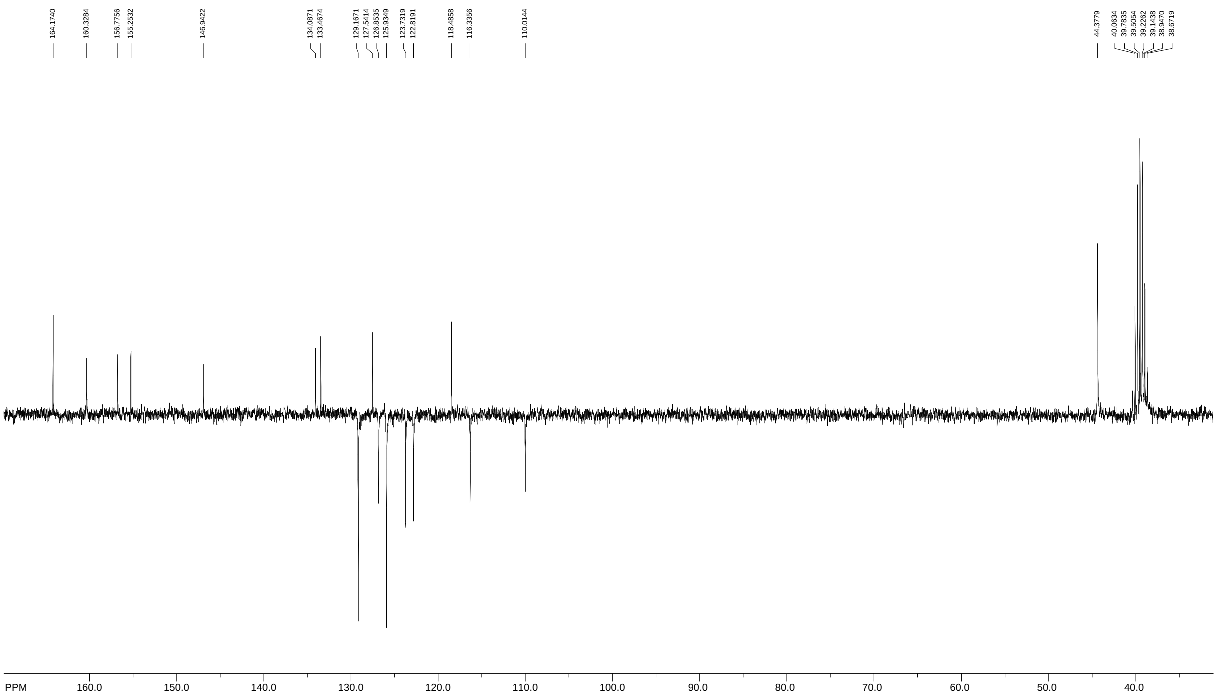

Figure S18. ^{13}C NMR spectrum (DMSO-d_6, 75 MHz) of 2-[5-(4-chlorophenyl)furan-2-yl]-6-(4,5-Dihydro-1*H*-imidazol-2-yl)benzothiazole hydrochloride **9**

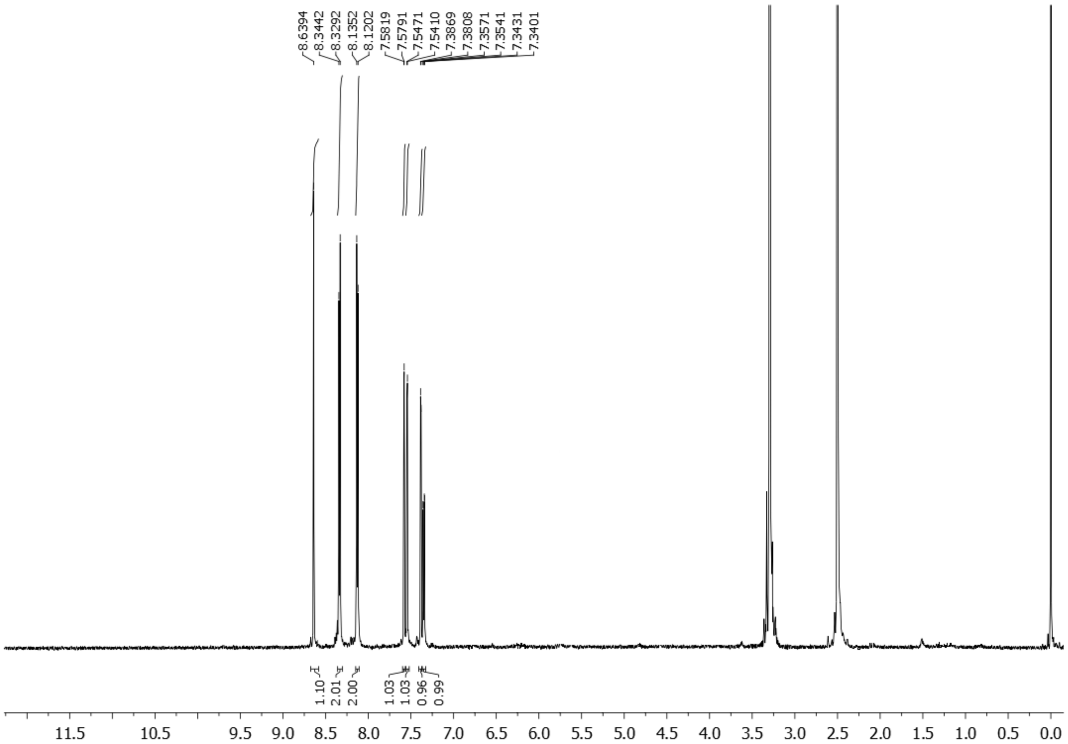

Figure S19. ^{1}H NMR spectrum (DMSO-d_6, 600 MHz) of 5(6)-cyano-2-[5-(4-nitrophenyl)furan-2-yl]benzimidazole **10**

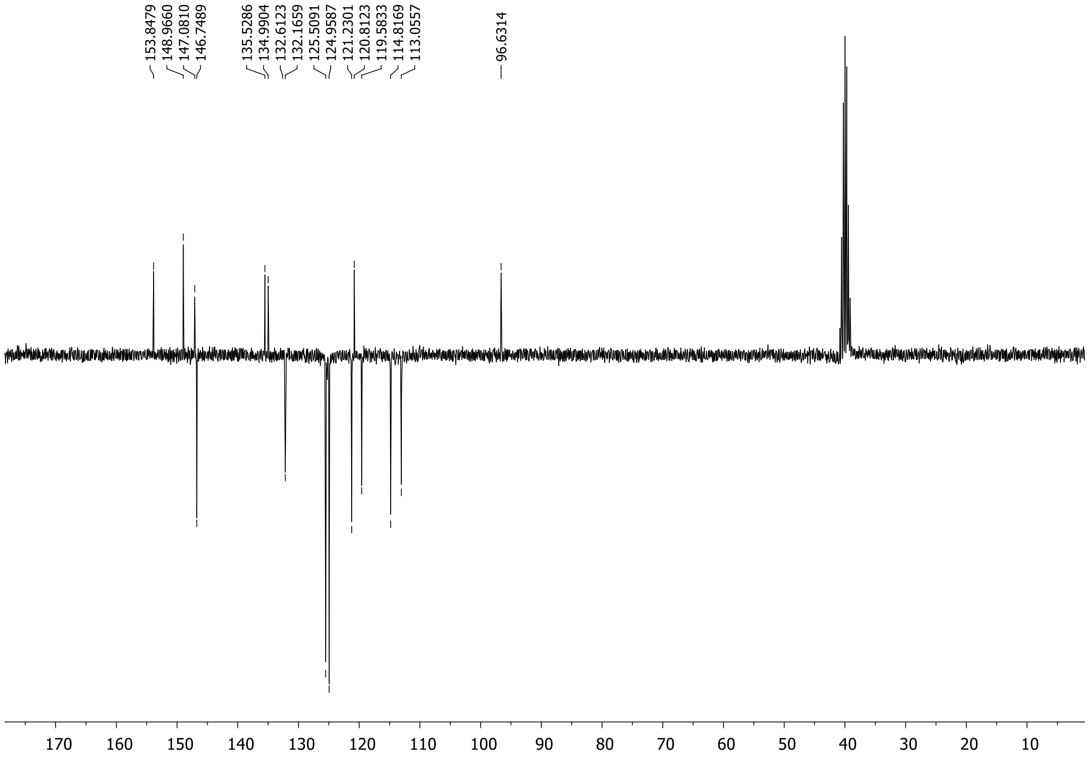

Figure S20. ^{13}C NMR spectrum (DMSO-d_6, 75 MHz) of 5(6)-cyano-2-[5-(4-nitrophenyl)furan-2-yl]benzimidazole **10**

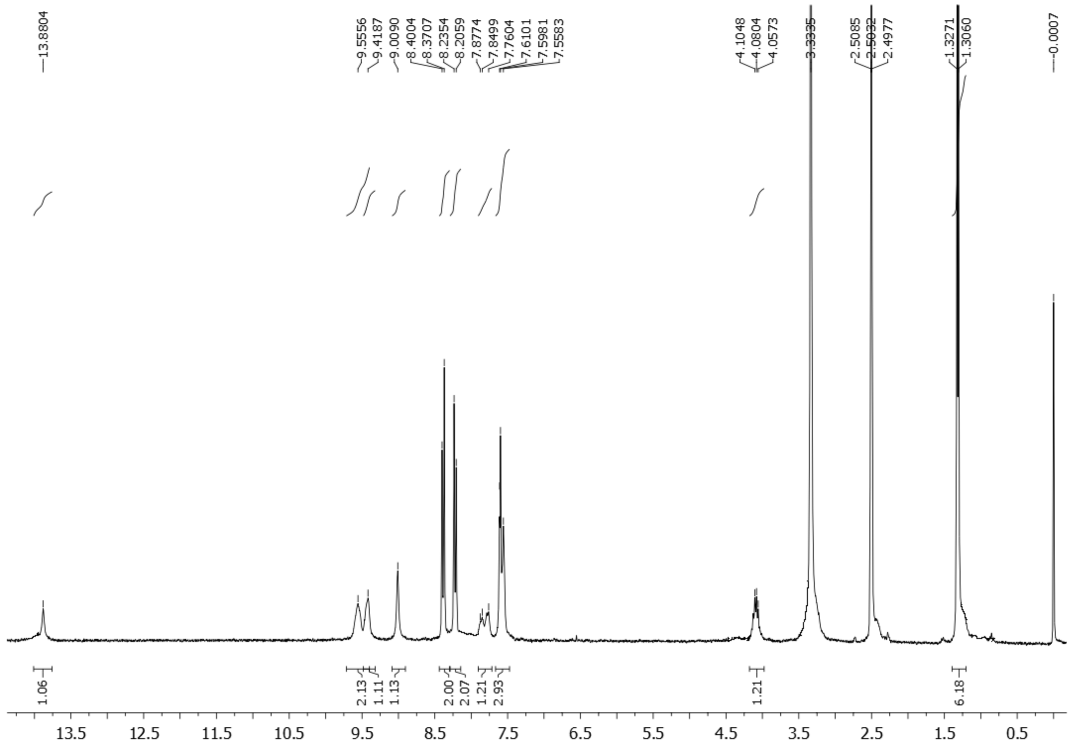

Figure S21. ^{1}H NMR spectrum (DMSO-d_6, 300 MHz) of 2-[5-(4-nitrophenyl)furan-2-yl]-5(6)-*N*-isopropilamidinobenzimidazole hydrochloride **11**

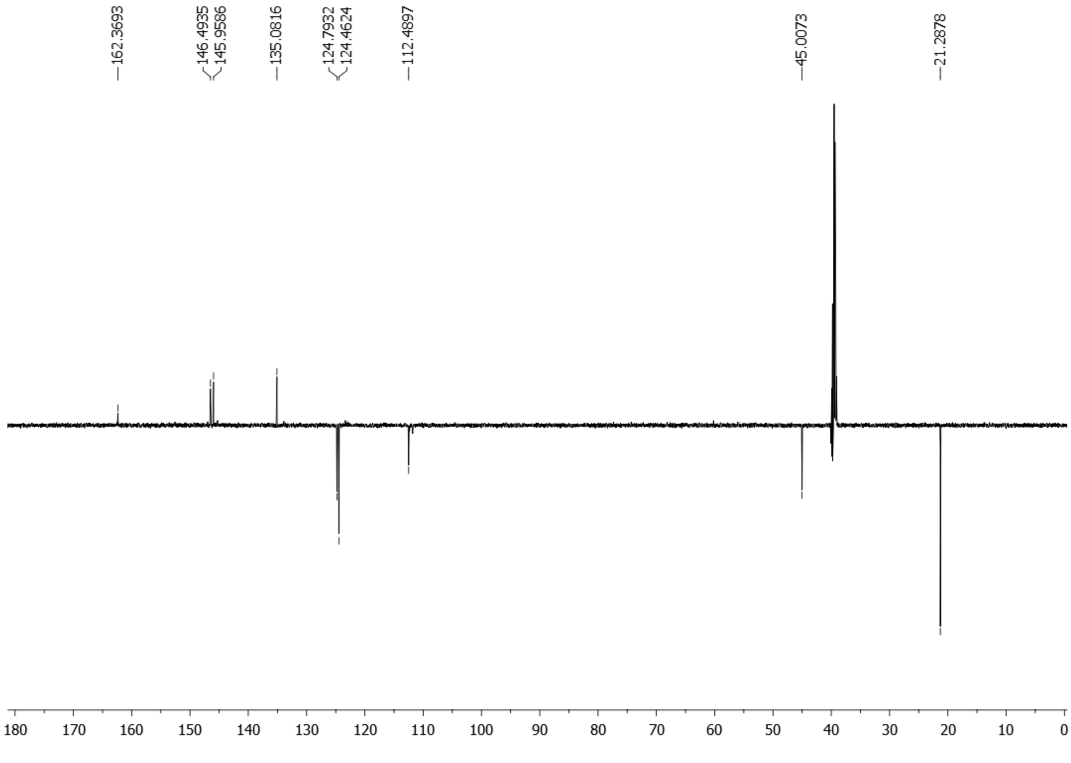

Figure S22. ^{13}C NMR spectrum (DMSO-d_6, 150 MHz) of 2-[5-(4-nitrophenyl)furan-2-yl]-5(6)-*N*-isopropilamidinobenzimidazole hydrochloride **11**

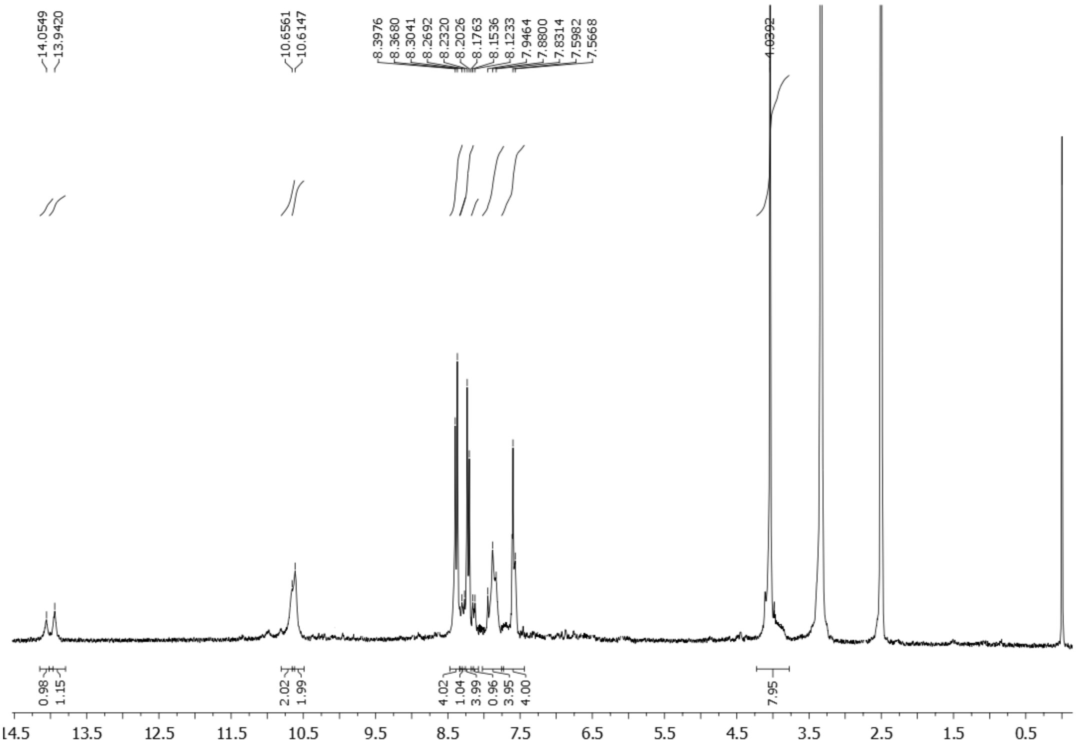

Figure S23. ^{1}H NMR spectrum (DMSO-d_6, 300 MHz) of 5(6)-(4,5-dihydro-1*H*-imidazol-2-yl)-2-[5-(4-nitrophenyl)furan-2-yl]benzimidazole hydrochloride **12**

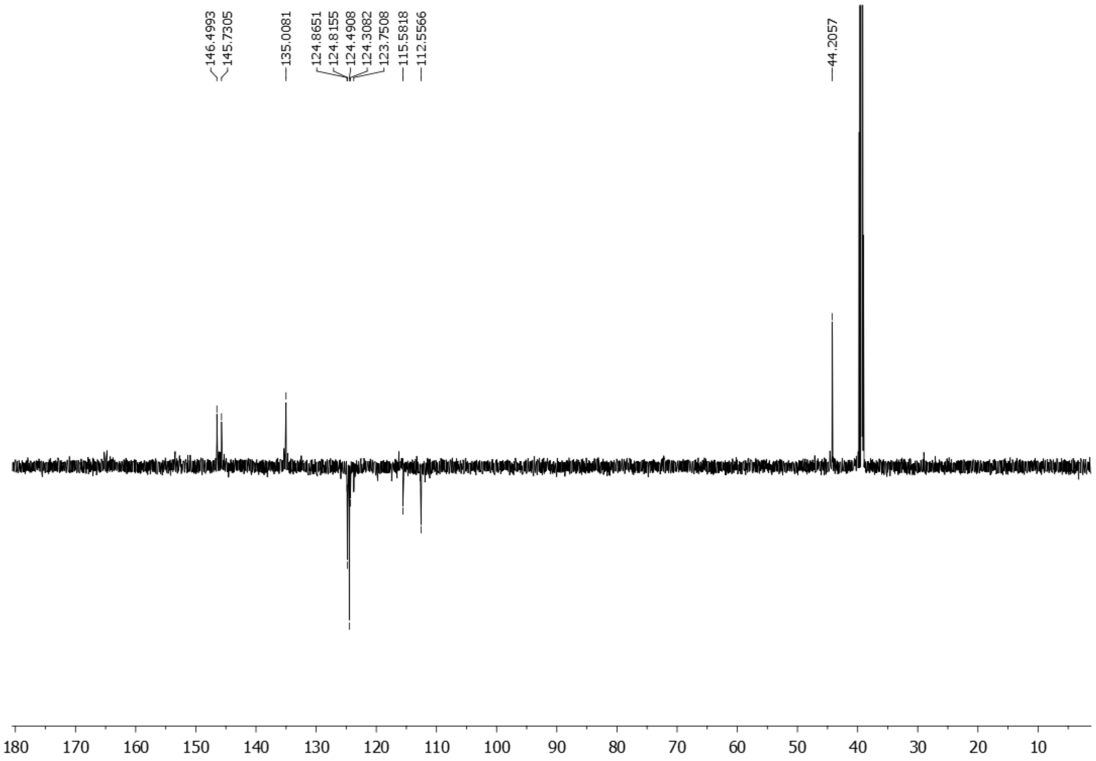

Figure S24. ^{13}C NMR spectrum (DMSO-d_6, 75 MHz) of 5(6)-(4,5-dihydro-1*H*-imidazol-2-yl)-2-[5-(4-nitrophenyl)furan-2-yl]benzimidazole hydrochloride **12**

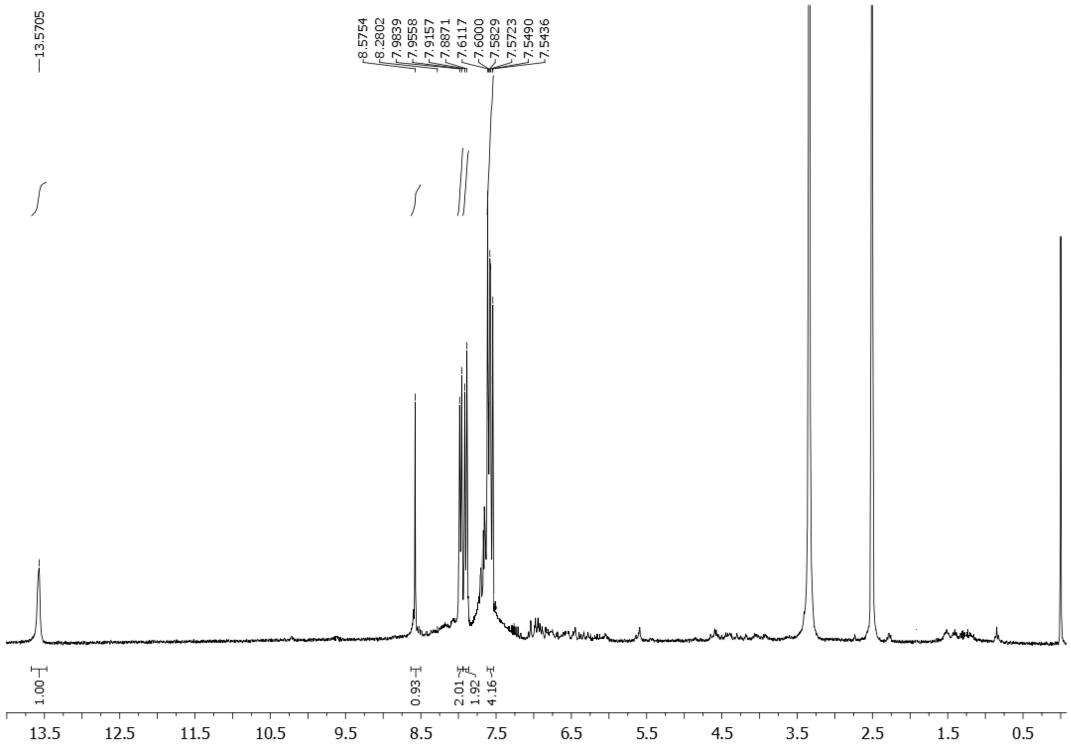

Figure S25. ^{1}H NMR spectrum (DMSO-d_6, 300 MHz) of 2-[5-(4-chlorophenyl)furan-2-yl]-5(6)-cyanobenzimidazole **13**

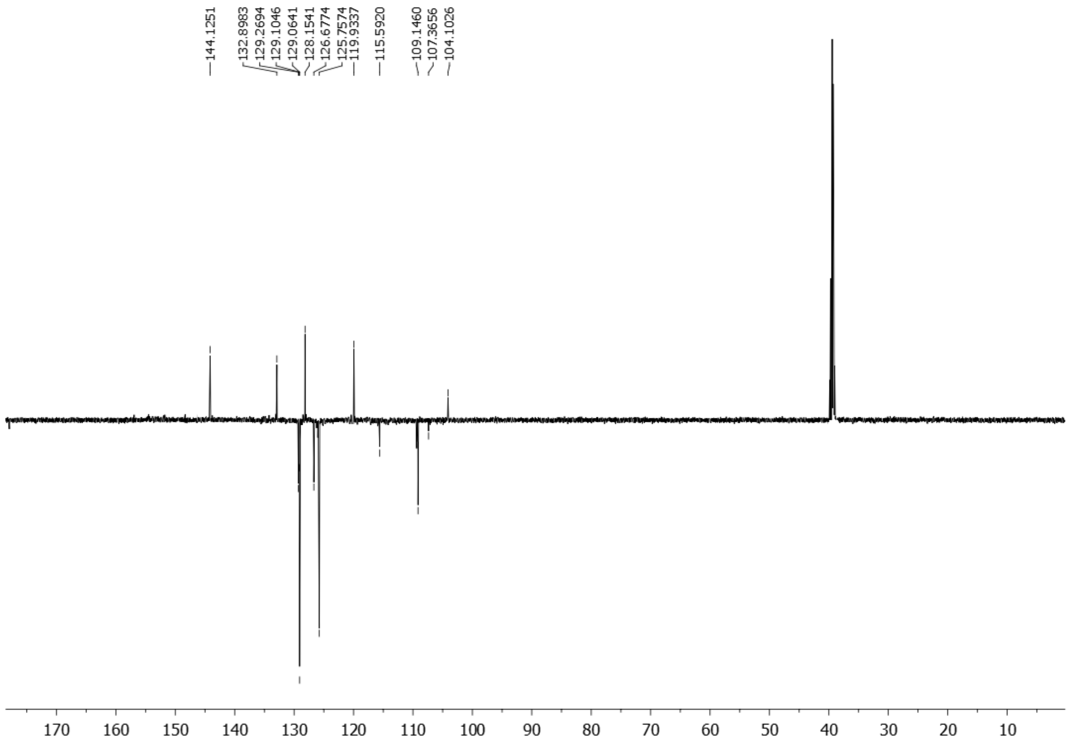

Figure S26. ^{13}C NMR spectrum (DMSO-d_6, 150 MHz) of 2-[5-(4-chlorophenyl)furan-2-yl]-5(6)-cyanobenzimidazole **13**

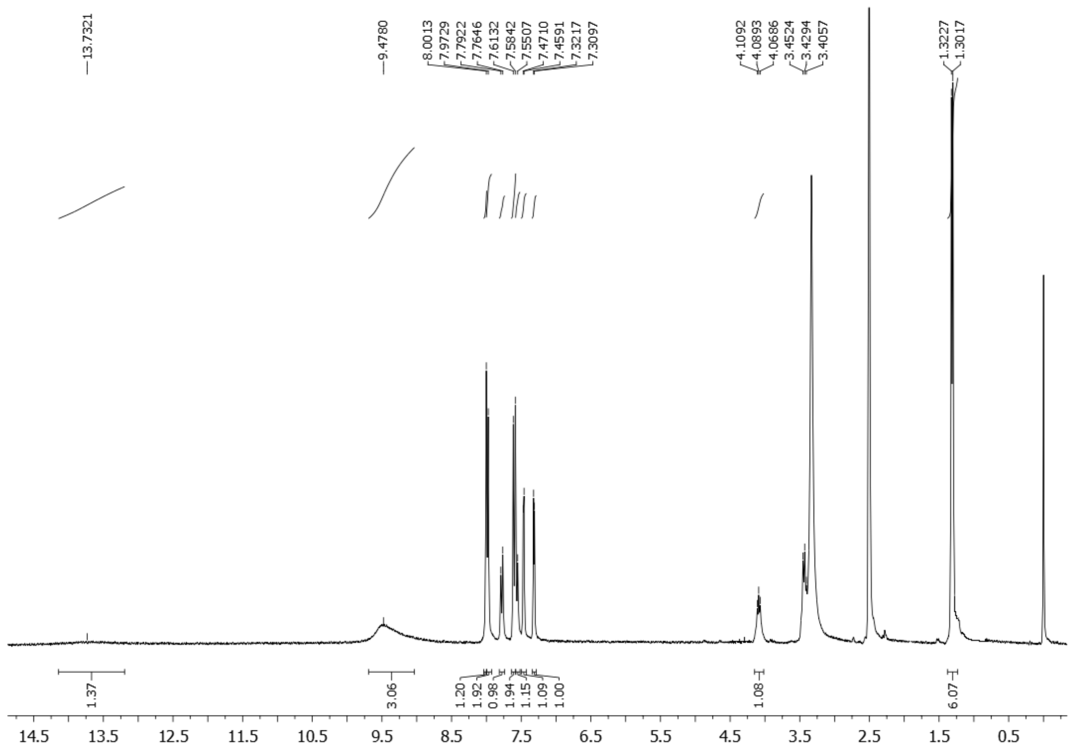

Figure S27. ^{1}H NMR spectrum (DMSO-d_6, 300 MHz) of 2-[5-(4-chlorophenyl)furan-2-yl]-5(6)-*N*-isopropilamidinobenzimidazole hydrochloride **14**

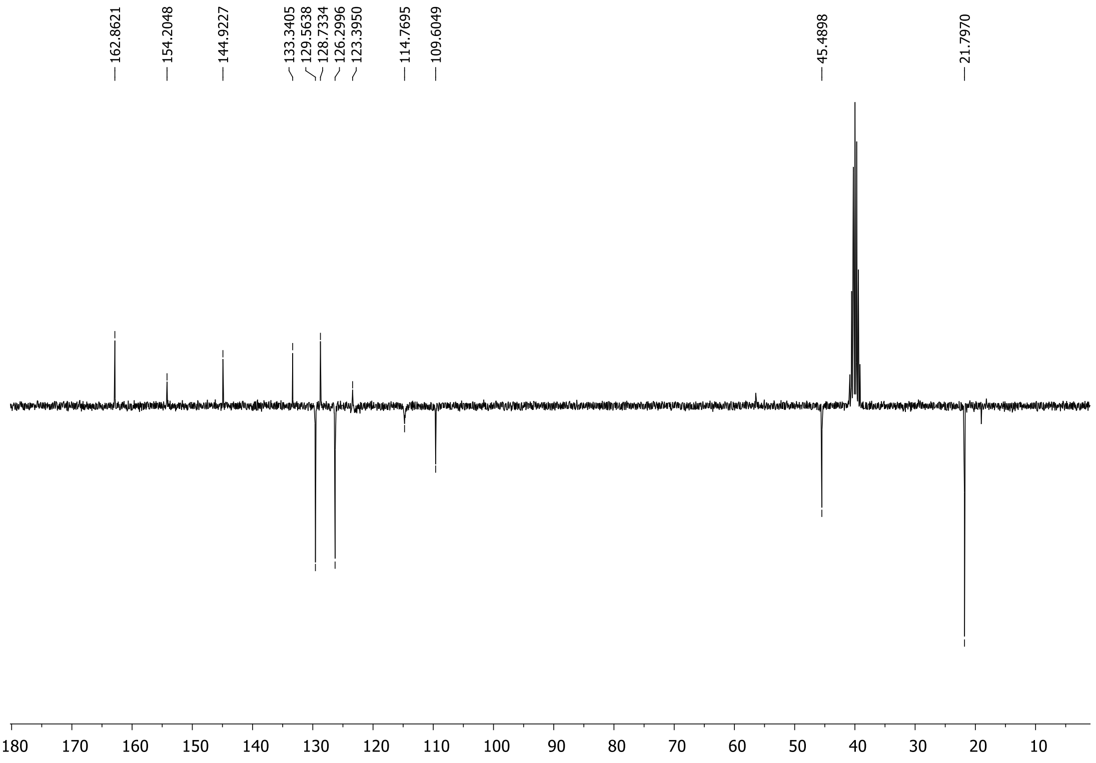

Figure S28. ^{13}C NMR spectrum (DMSO-d_6, 75 MHz) of 2-[5-(4-chlorophenyl)furan-2-yl]-5(6)-*N*-isopropilamidinobenzimidazole hydrochloride **14**

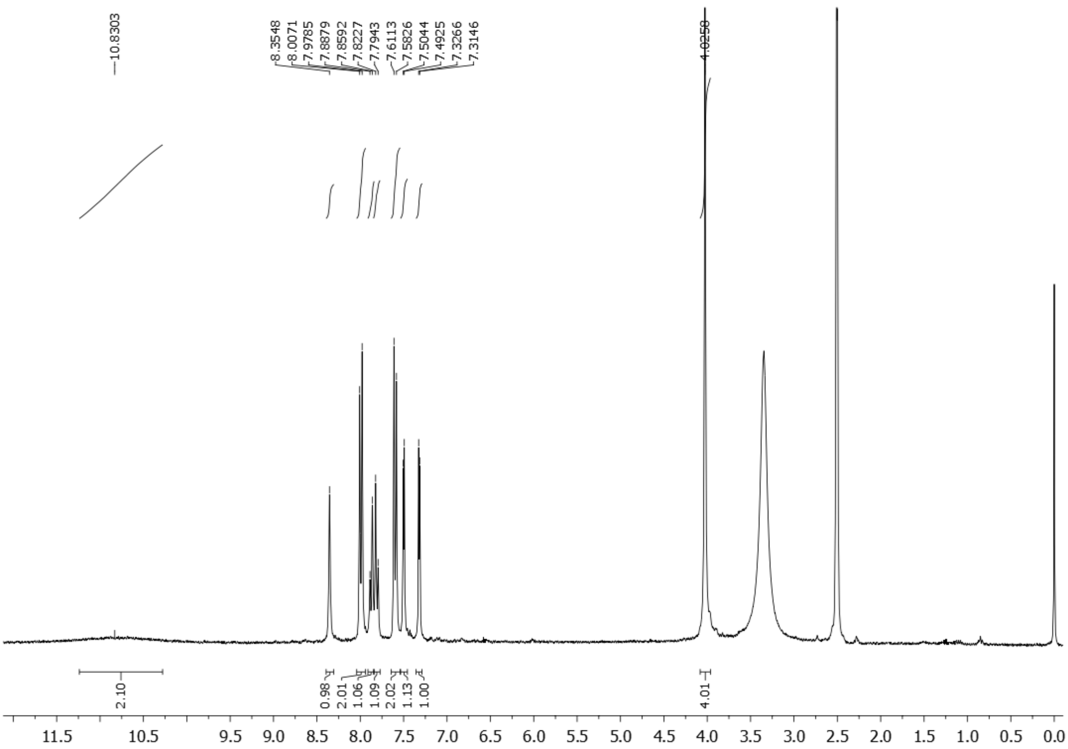

Figure S29. ^{1}H NMR spectrum (DMSO-d_6, 300 MHz) of 2-[5-(4-chlorophenyl)furan-2-yl]-5(6)-(4,5-dihydro-1*H*-imidazol-2-yl)benzimidazole hydrochloride **15**

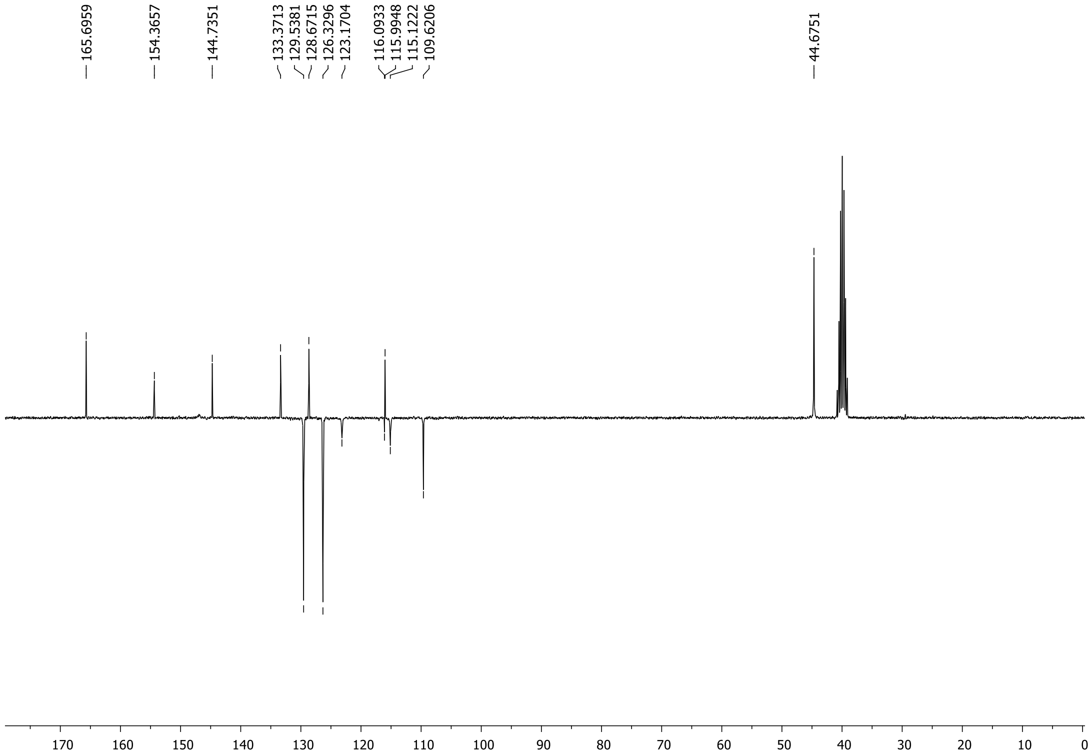

Figure S30. ^{13}C NMR spectrum (DMSO-d_6, 75 MHz) of 2-[5-(4-chlorophenyl)furan-2-yl]-5(6)-(4,5-dihydro-1*H*-imidazol-2-yl)benzimidazole hydrochloride **15**

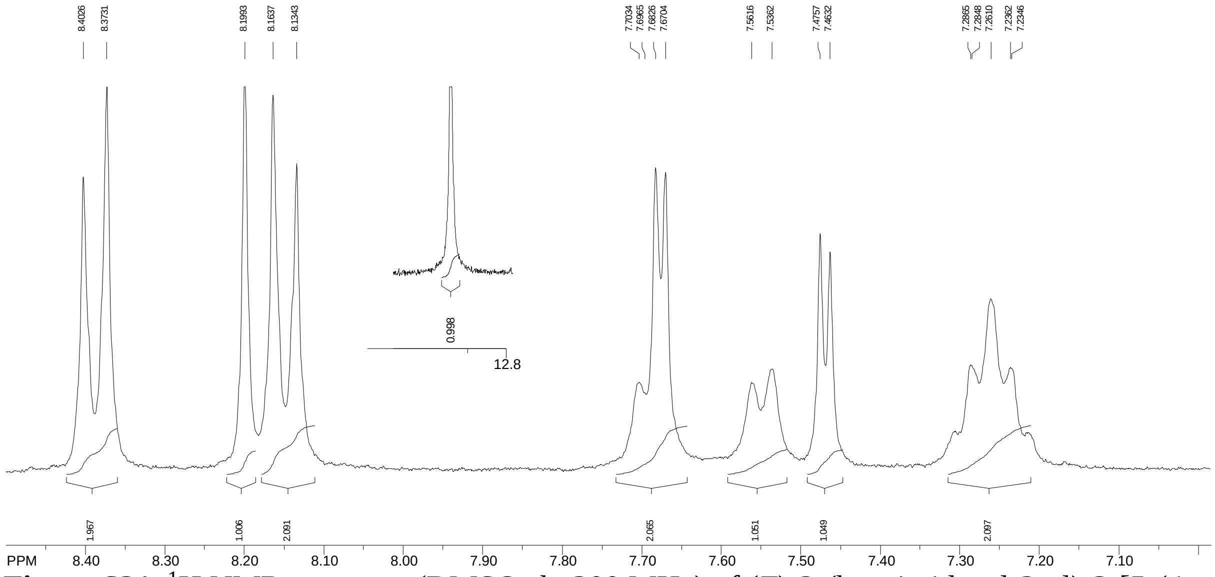

Figure S31. ^{1}H NMR spectrum (DMSO-d_6, 300 MHz) of (*E*)-2-(benzimidazol-2-yl)-3-[5-(4-nitrophenyl)furan-2-yl]acrylonitrile **17**

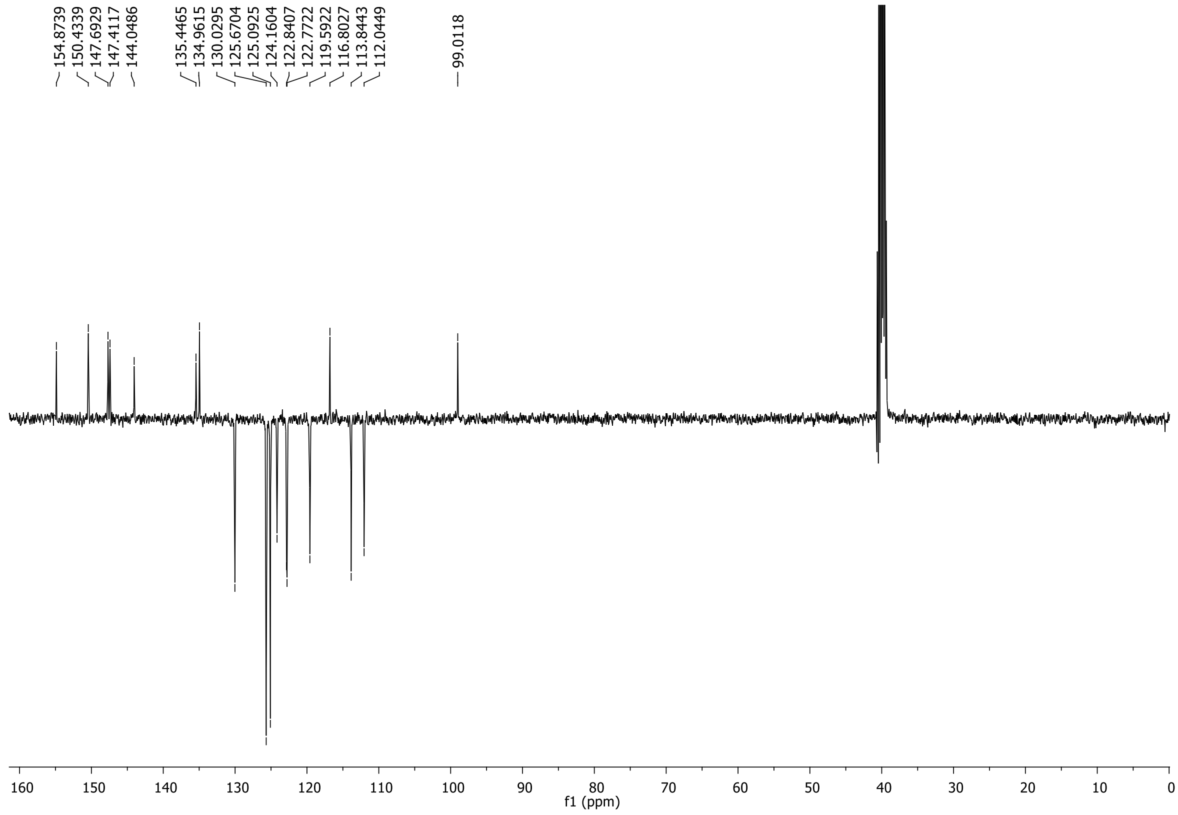

Figure S32. ^{13}C NMR spectrum (DMSO-d_6, 300 MHz) of (*E*)-2-(benzimidazol-2-yl)-3-[5-(4-nitrophenyl)furan-2-yl]acrylonitrile **17**

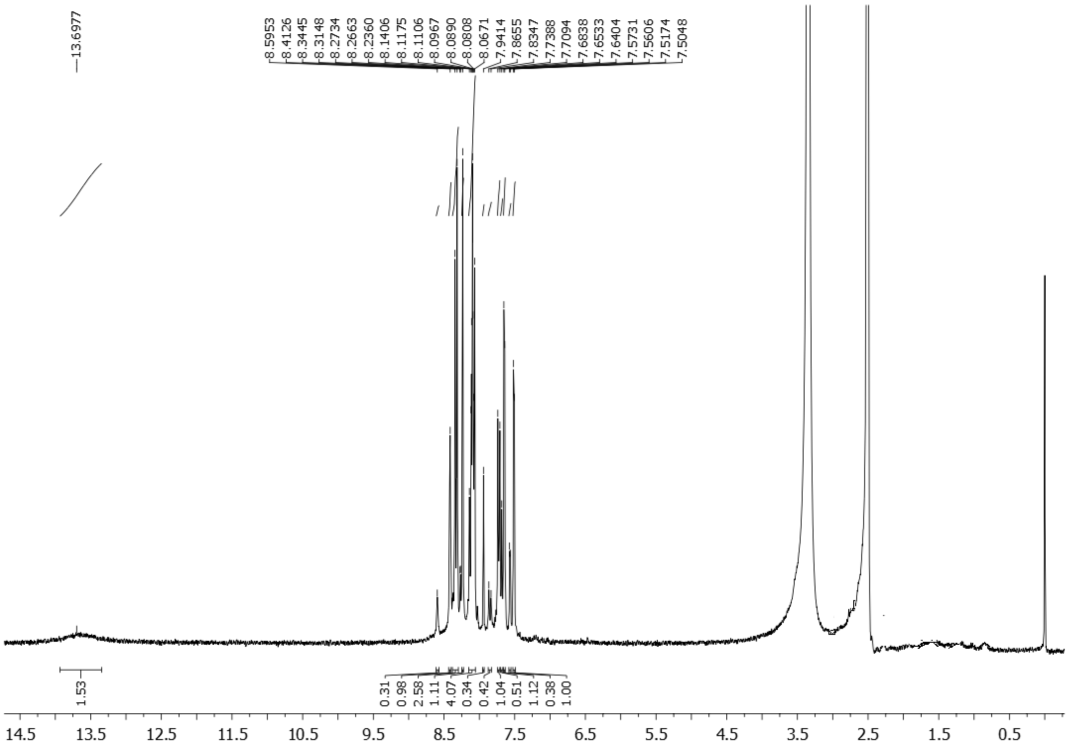

Figure S33. ^{1}H NMR spectrum (DMSO-d_6, 300 MHz) of (*E*)-2-(5(6)-nitrobenzimidazol-2-yl)-3-[5-(4-nitrophenyl)furan-2-yl]acrylonitrile **18**

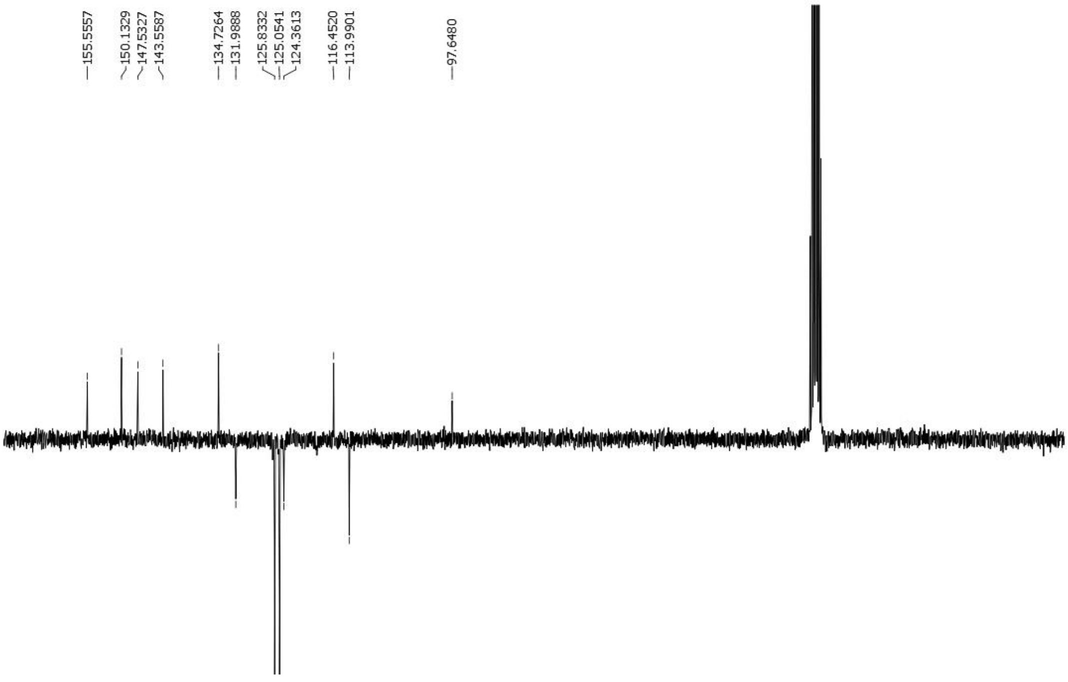

Figure S34. ^{13}C NMR spectrum (DMSO-d_6, 75 MHz) of (*E*)-2-(5(6)-nitrobenzimidazol-2-yl)-3-[5-(4-nitrophenyl)furan-2-yl]acrylonitrile **18**

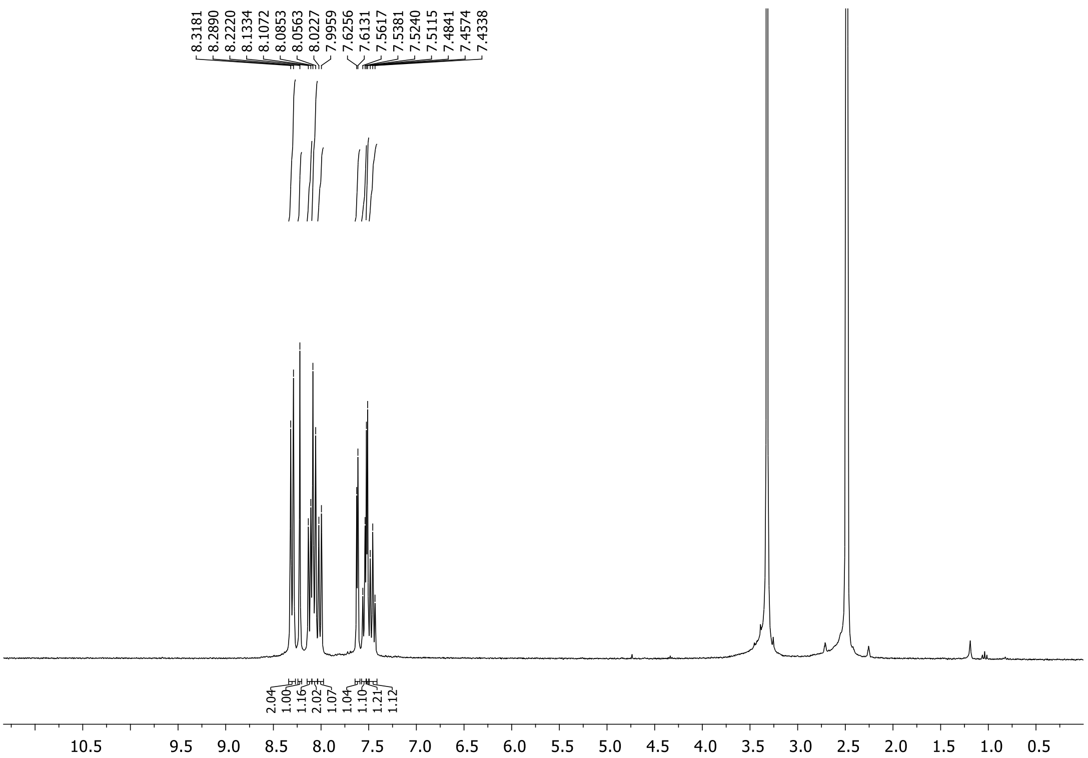

Figure S35. ^{1}H NMR spectrum (DMSO-d_6, 300 MHz) of (*E*)-2-(benzothiazol-2-yl)-3-[5-(4-nitrophenyl)furan-2-yl]acrylonitrile **19**

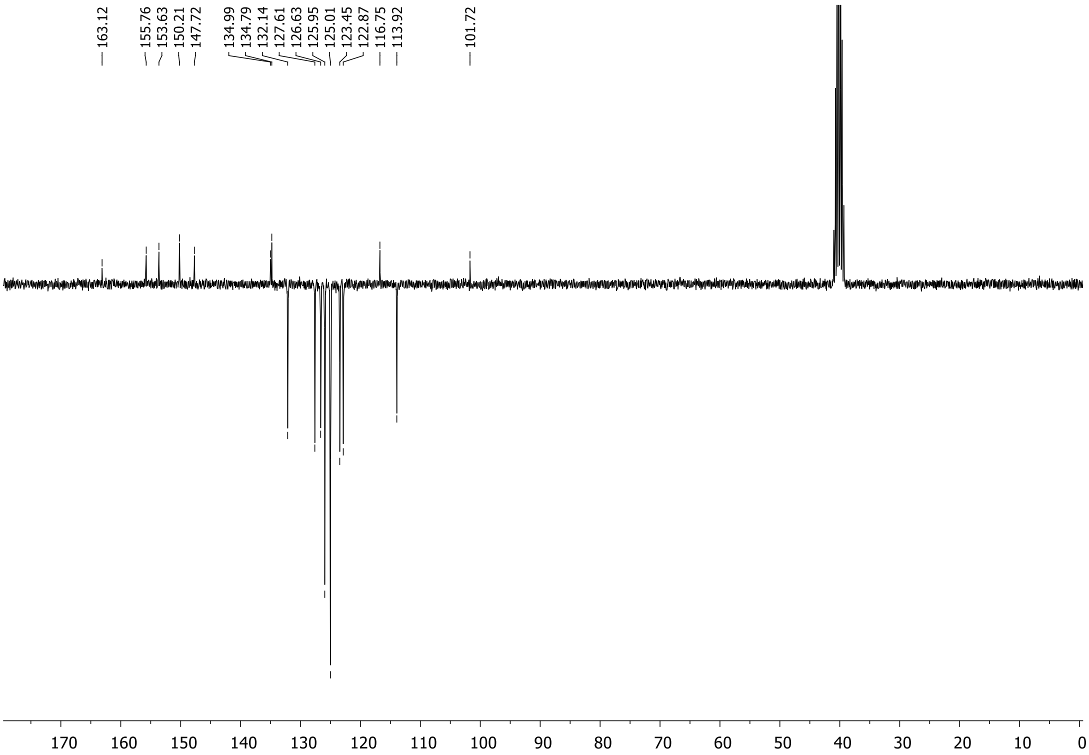

Figure S36. ^{13}C NMR spectrum (DMSO-d_6, 75 MHz) of**.** (*E*)-2-(benzothiazol-2-yl)-3-[5-(4-nitrophenyl)furan-2-yl]acrylonitrile **19**

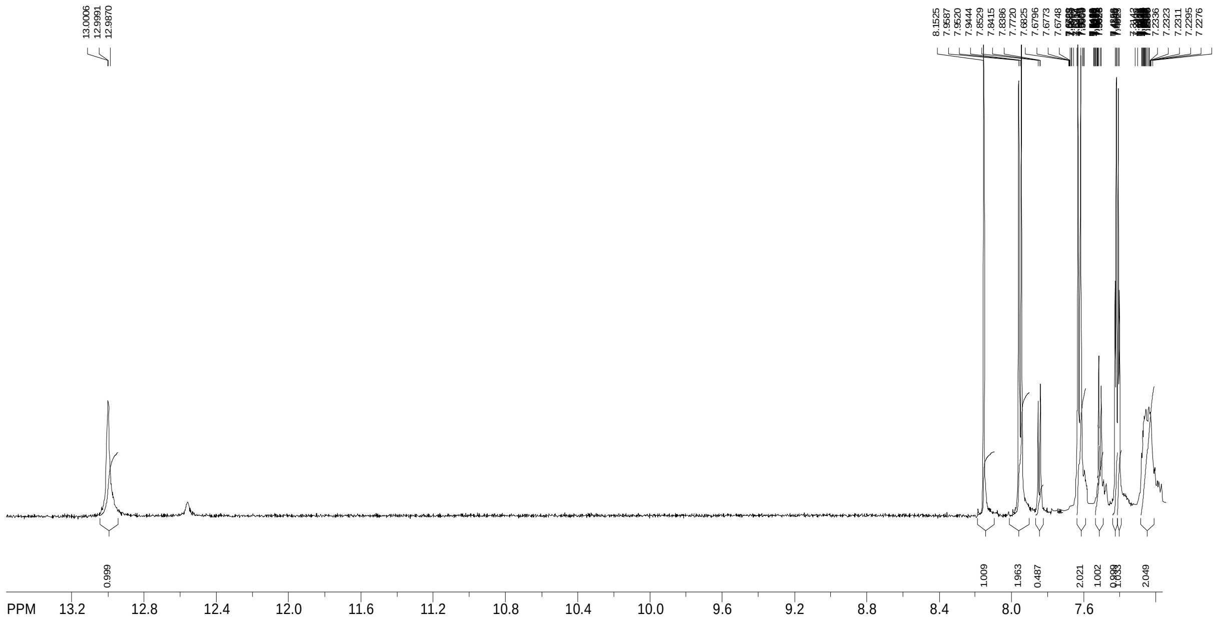

Figure S37. ^{1}H NMR spectrum (DMSO-d_6, 300 MHz) of (*E*)-2-(benzimidazol-2-yl)-3-[5-(4-chlorophenyl)furan-2-yl]acrylonitrile **20**

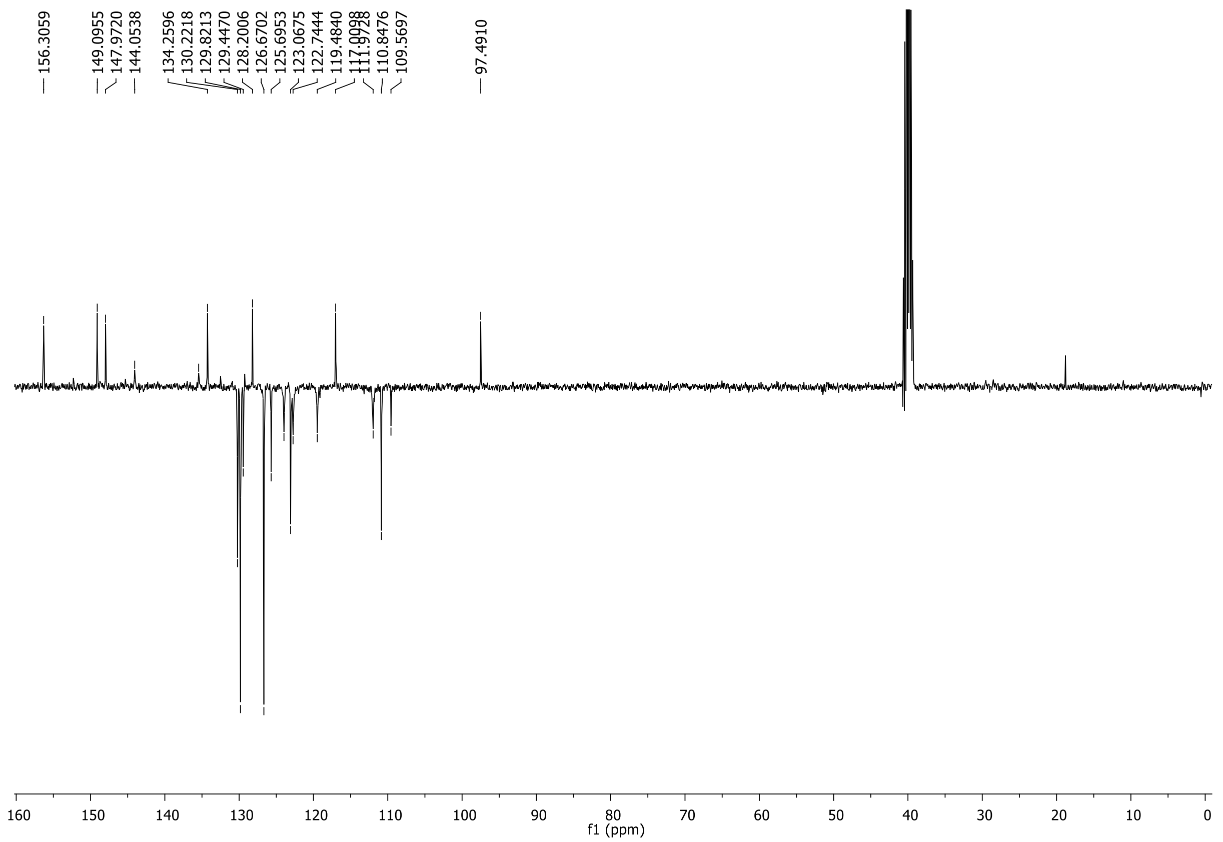

Figure S38. ^{13}C NMR spectrum (DMSO-d_6, 300 MHz) of (*E*)-2-(benzimidazol-2-yl)-3-[5-(4-chlorophenyl)furan-2-yl]acrylonitrile **20**

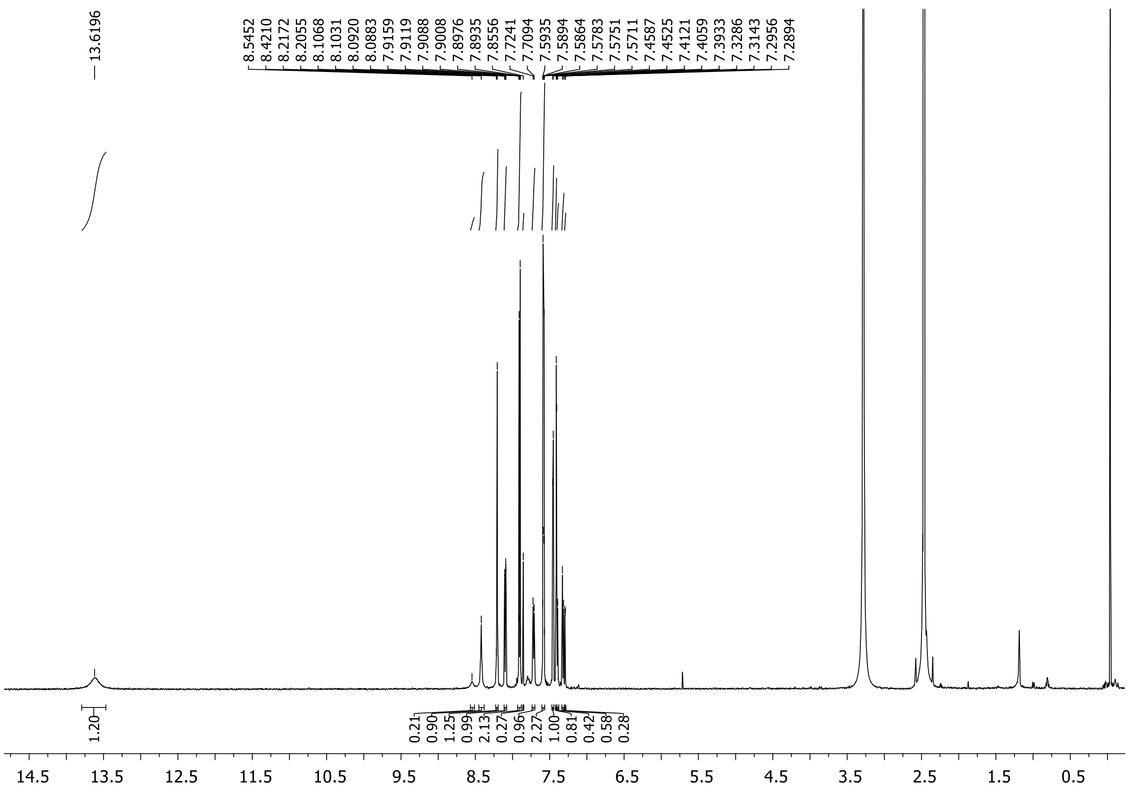

Figure S39. ^{1}H NMR spectrum (DMSO-d_6, 600 MHz) of (*E*)-3-[5-(4-chlorophenyl)furan-2-yl]-2-(5(6)-nitrobenzimidazol-2-yl)acrylonitrile **21**

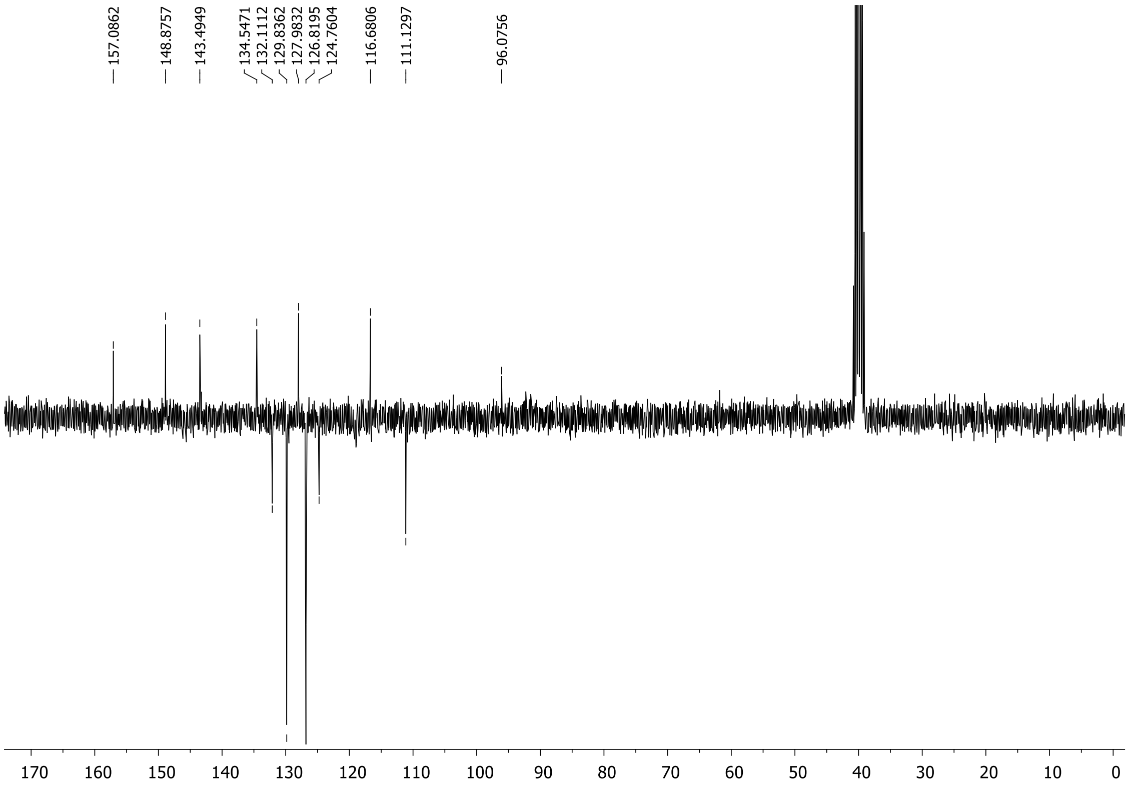

Figure S40. ^{13}C NMR spectrum (DMSO-d_6, 75 MHz) of (*E*)-3-[5-(4-chlorophenyl)furan-2-yl]-2-(5(6)-nitrobenzimidazol-2-yl)acrylonitrile **21**

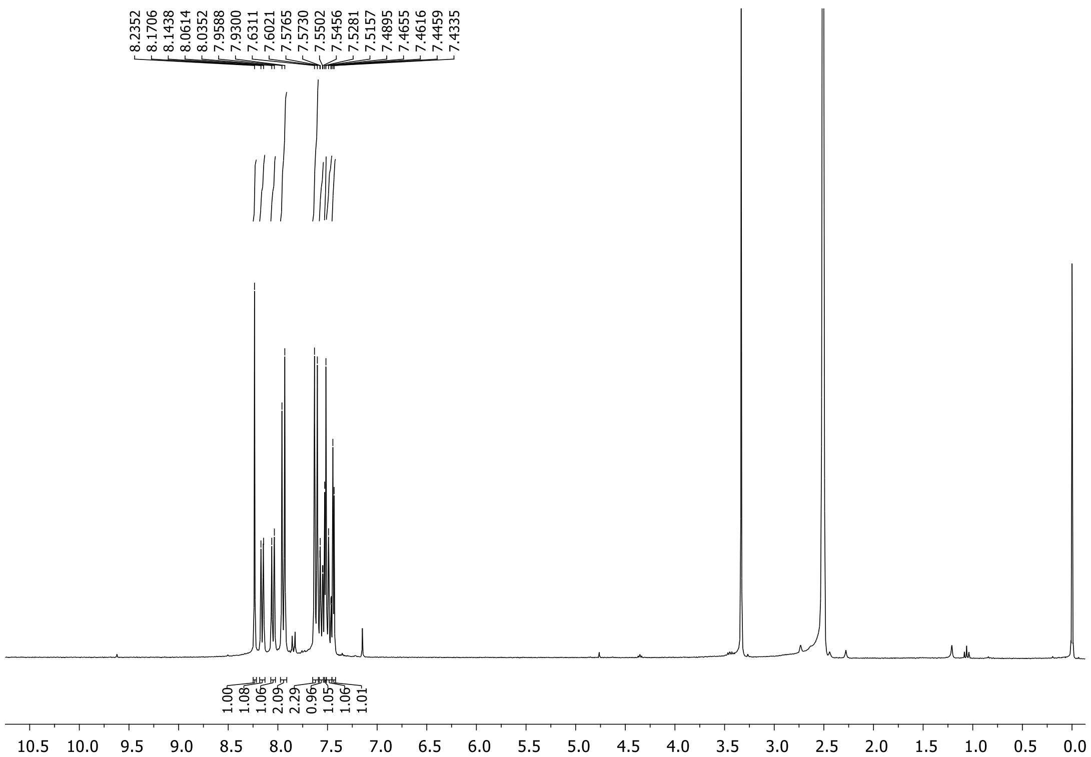

Figure S41. ^{1}H NMR spectrum (DMSO-d_6, 300 MHz) of (*E*)-2-(benzothiazol-2-yl)-3-[5-(4-chlorophenyl)furan-2-yl]acrylonitrile **22**

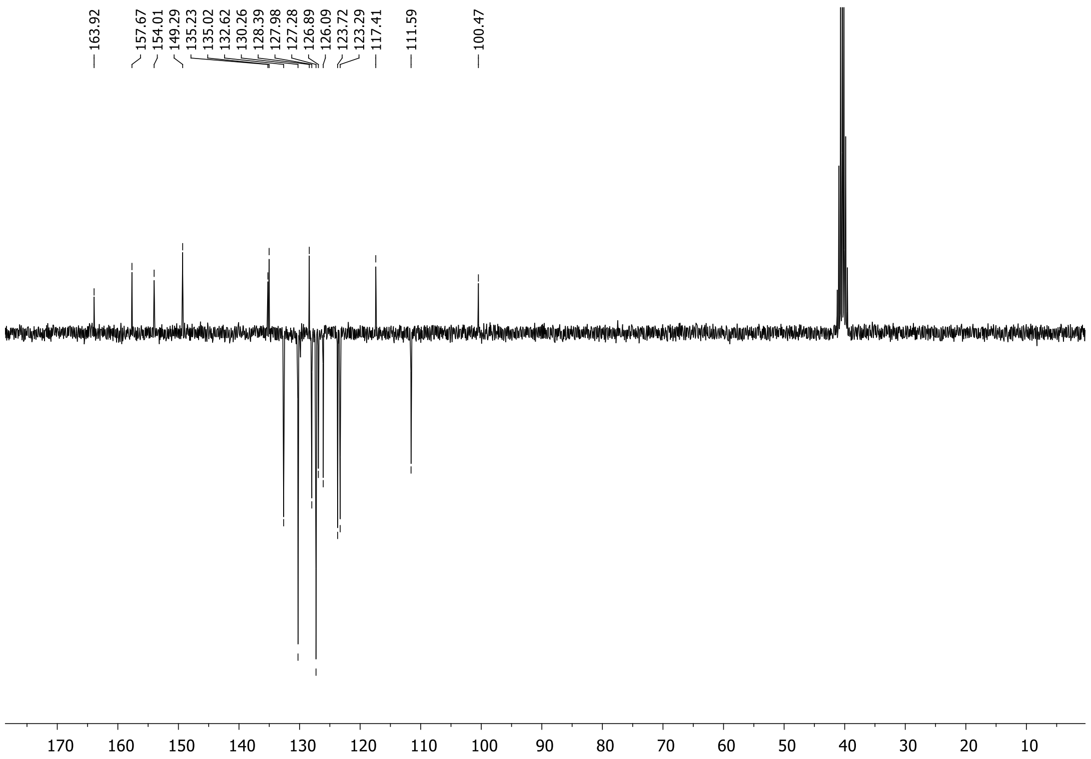

Figure S42. ^{13}C NMR spectrum (DMSO-d_6, 75 MHz) of (*E*)-2-(benzothiazol-2-yl)-3-[5-(4-chlorophenyl)furan-2-yl]acrylonitrile **22**
